# Diversity and Correlation Analysis of Differential Amino Acid Metabolites and Dominant Endophytic Bacteria in *Lycium chinense* Fruits at Different Stages

**DOI:** 10.3390/genes16070836

**Published:** 2025-07-18

**Authors:** Chongxin Yin, Huichun Xie, Xiaoli Yang, Lianyu Zhou, Guigong Geng, Feng Qiao

**Affiliations:** 1 Key Laboratory of Tibetan Plateau Medicinal Plant and Animal Resources, School of Life Sciences, Qinghai Normal University, Xining 810008, China; yinchongxin0701@163.com (C.Y.); yezino.1@163.com (H.X.); zly7604@163.com (L.Z.); 2 Academy of Plateau Science and Sustainability, Qinghai Normal University, Xining 810008, China; 3 Qinghai Provincial Key Laboratory of Plateau Climate Change and Corresponding Ecological and Environmental Effects, Qinghai Institute of Technology, Xining 810016, China; xlyang@qhit.edu.cn; 4 Academy of Agricultural and Forestry Sciences, Qinghai University, Xining 810016, China; genggg-298@163.com

**Keywords:** *Lycium chinense*, fruits, amino acid metabolites, endophytic bacteria, correlation analysis

## Abstract

**Background**: *Lycium* *chinense* has been acknowledged for its substantial nutritional benefits. The “Mengqi No.1” variety of *L. chinense* is known for its high yield and exceptional quality. **Methods**: We screened twenty dominant endophytic bacterial genera based on OTUs from *L. chinense* fruits during three developmental stages. **Results**: Forty-three differential amino acid metabolites were selected from *L. chinense* fruits. Five endophytic bacteria (*Enterococcus*, *Escherichia-Shigella*, *Bacteroides*, *Pseudomonas*, and *Bacillus)* were dominant genera in green fruit (GF, 16–19 days after flowering), color-changing fruit (CCF, 22–25 days after flowering), and red-ripe fruit (RRF, 31–34 days after flowering). Four endophytic bacterial genera (*Enterococcus*, *Bacillus*, *Pseudomonas*, and *Rhodanobacter*) showed positive correlation with twenty different amino acid metabolites and negative correlation with seven different amino acid metabolites. **Conclusions**: Five genes (*AST1*, *ltaE1*, *TAT1*, *SHMT2*, and *SHMT3*) indicated positive correlation with seventeen different amino acid metabolites and negative correlation with eight different amino acid metabolites. *AST1 *gene had a major role in regulating arginine biosynthesis (ko00220); *ltaE1*, *SHMT2,* and *SHMT3* genes were major in regulating glycine, serine, and threonine metabolism (ko00260); and *TAT1* gene had a major role in regulating tyrosine metabolism (ko00350). These findings offer insights into the relationship between amino acid synthesis and endophytic bacteria in *L. chinense* fruits.

## 1. Introduction

Amino acids serve as key constituents in the metabolism of proteins, energy, and nitrogen within the primary metabolic processes of the plant. They act as precursors for a range of active compounds that play specific roles in plant–microbe interactions, which remain largely unexplored. It is probable that individual amino acids also participate in signaling events that occur during biotic interactions [1]. Camalexin, a sulfur-containing indolic compound with antifungal properties, is derived from tryptophan (Trp). This compound is particularly specific to the Brassicaceae family and serves as the most significant phytoalexin found in Arabidopsis [2]. Despite concentrating on amino acid-derived compounds, the specialized metabolism of plants is exceedingly intricate and possesses the capability to offer a significant degree of specificity in plant–microbe interactions [1]. Altering the structural arrangement of amino acids served as a strategy to prevent the microbial breakdown of these compounds [3]. Lysine acetylation could play a significant regulatory role in modulating the function of aminoacyl-tRNA synthetases (aaRSs) and the synthesis of proteins [4]. The process of amino acid exudation by plants necessitates transport across multiple membranes, including the apoplast and cytoplasm for exudation or uptake, as well as across the membranes of intracellular compartments involved in the synthesis, metabolism, and storage of amino acids, such as chloroplasts, mitochondria, and vacuoles [5]. Additionally, this transport occurs between various cells and plant organs to fulfill the heightened local demand resulting from interactions with microbes [1].

Endophytes that inhabit various parts of plants, including roots, stems, flowers, and fruits, obtain nutrients from their host and were well known for their mutualistic associations with host plants. They play a crucial role in enhancing plant growth and assisting in the management of abiotic stress [6]. Mycorrhizal fungi and endophytes associated with plants significantly contribute to plant health by facilitating the acquisition of essential nutrients and managing environmental stressors [7,8,9]. Both plants and microbes synthesize a variety of diverse metabolites and proteins that serve multiple functions within the context of organismal and environmental interactions [10]. The enhancement of plant growth and fitness through endophytes underscores their potential as environmentally friendly alternatives for increasing crop production while reducing reliance on synthetic fertilizers and pesticides [7], provided that current limitations in commercial applications were addressed [10]. In addition to their essential ecological functions, endophytes were increasingly recognized as valuable biological resources for the production of high-value metabolites with pharmacological significance. These organisms exhibit host mimicry through their autonomous synthesis of metabolites, which were anticipated to be promising candidates for genetic engineering applications [11].

The use of *L. chinense* has been widespread in addressing a range of health issues [12]. *Lycium barbarum* polysaccharide (LBP) has been widely reported to exhibit anti-aging, antioxidant, anti-apoptotic, and anti-inflammatory effects [13,14]. It has demonstrated significant potential in the prevention and treatment of various diseases, including adolescent depression, neuroprotection, retinal protection, and heart failure [15,16,17,18]. The vitamin C, phenols, flavonoids, and carotenoids present in *L. chinense* were intrinsically linked to their nutritional and health-promoting properties [13,19,20]. The extract derived from *Lycium barbarum* has been shown to be positively associated with anti-inflammatory responses by suppressing the expression of interleukin (IL)-1β and tumor necrosis factor (TNF)-α [21], as well as mitigating lipopolysaccharide (LPS)-induced inflammation [22]. Additionally, it exhibits antioxidant properties that may aid in the management of Parkinson’s disease, with protective effects on neurons noted in studies related to retinal ischemia and reperfusion damage [23,24]. Research indicates that goji berries were often combined with fruits such as jujube, black sesame, and walnuts to create gelatinous cakes, which provide considerable benefits for mental well-being and treatments for blood deficiency and anemia-related issues [25,26].

Research on amino acid metabolism and endophytic bacteria in *L. chinense* fruit remains limited. This study investigates the types and changes of amino acid metabolites during the development of *L. chinense* fruit and analyzes the dominant endophytic bacteria present. The results provide a scientific basis for understanding the relationship between endophytic bacteria and amino acid metabolism in *L. chinense* fruit.

## 2. Materials and Methods

### 2.1. Plant Materials

**Figure 1:**
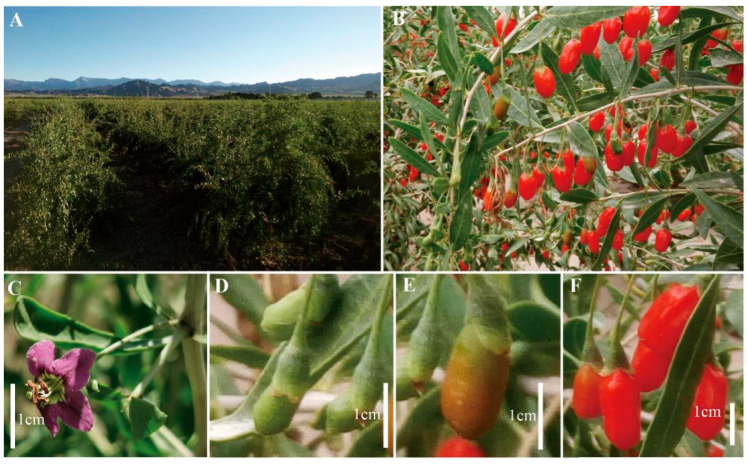
Phenological stages and collection sites of *L. chinense* fruits. (**A**) Cultivation location: Nuomuhong Farm, Qaidam Basin (36°23′26.84″ N, 94°26′49.04″ E; altitude: 2745 m, Qinghai, China). (**B**) Fruit morphology. (**C**) Flowering phase. (**D**) Green fruit (GF, 16–19 days post-anthesis). (**E**) Color-changing fruit (CCF, 22–25 days post-anthesis). (**F**) Red-ripe fruit (RRF, 31–34 days post-anthesis). Scale bar: 1 cm.

The cultivation of the *L. chinense* variety ‘Mengqi No.1’ occurred at Nuomuhong Farm in China’s Qinghai Province, which lies within the Qaidam Basin (36°23′26.84″ N, 94°26′49.04″ E; altitude: 2745 m). Characterized by aridity, prolonged sunlight exposure, and significant diurnal temperature fluctuations (peak: 35.8 °C; minimum: −31 °C), this region receives approximately 58.51 mm of annual precipitation. Fruit maturation following flowering and fertilization lasts 28–35 days. As illustrated in Figure 1, three distinct developmental phases define *L. chinense* fruit progression: green fruit (GF, 16–19 days post-flowering), color-changing fruit (CCF, 22–25 days post-flowering), and red-ripe fruit (RRF, 31–34 days post-flowering). All collected fruits exhibited uniform dimensions, full maturity, and no disease or pest damage. Liquid nitrogen rapidly froze specimens, which were subsequently stored at −80 °C for further analysis. Four duplicate samples collected from each fruit, GF, CCF, and RRF, for a total of twelve samples, were used for subsequent analysis.

### 2.2. Preparation of Samples from the Fruits of L. chinense

Fruit samples of *L. chinense* at three developmental stages were collected and subjected to vacuum freeze-drying. Precisely 50 mg aliquots were then mixed with 1000 μL of extraction solution (methanol:acetonitrile:water = 2:1:1). Following homogenization at 45 Hz for 10 min using a grinder, samples underwent ice-bath sonication for an additional 10 min, subsequently rested at −20 °C for 1 h, and were centrifuged (4 °C, 12,000× *g*, 10 min). From the resulting supernatant, 500 μL was vacuum-concentrated, reconstituted in 160 μL of 50% acetonitrile, and vortex-mixed thoroughly. After a second ice-bath incubation (10 min) and re-centrifugation (identical conditions), 120 μL of supernatant was transferred to 2 mL injection vials. A pooled QC sample, generated by combining 10 μL from each sample, was prepared for instrumental analysis.

### 2.3. LC-MS/MS Analysis

Liquid chromatography-tandem mass spectrometry (LC-MS/MS) was conducted utilizing a Whatsch Acquisition I-Class PLUS ultra-high-performance liquid chromatography (UHPLC) system coupled to an AB Sciex Qtrap 6500+ mass spectrometer, renowned for exceptional sensitivity. Chromatographic conditions were set as follows: a Waters Acquisition UPLC HSS-T3 column (1.8 µm, 2.1 mm × 100 mm) served as the stationary phase. The mobile phase comprised Phase A: ultrapure water containing 0.1% formic acid and 5 mM ammonium acetate; and Phase B: acetonitrile supplemented with 0.1% formic acid.

For gradient elution, the initial composition (98% A: 2% B) was maintained for 1.5 min, followed by a linear shift to 50% A/50% B over 5 min. Subsequently, the gradient transitioned to 2% A/98% B within 9 min, held for 1 min, before reverting to initial conditions (98% A/2% B) in 1 min, with equilibration extended for an additional 3 min. Operational parameters included a constant flow rate (350 μL/min) and column temperature (50 °C). Effluent was directed to an ESI-triple quadrupole-linear ion trap (QTRAP) mass spectrometer for detection.

Electrospray ionization (ESI) source parameters were configured as follows: the temperature was maintained at 550 °C; and ion spray voltage (IS) was set to 5500 V (positive mode) or −4500 V (negative mode). Curtain gas (CUR: 35 psi) and ionization gases (GSI: 50 psi; GSII: 55 psi) operated at specified pressures, with collision-activated dissociation (CAD) at medium intensity. Instrument calibration in both QQQ and LIT modes employed polypropylene glycol solutions (10 and 100 μmol/L, respectively). For QQQ scans, MRM experiments utilized nitrogen as collision gas (medium level), while declustering potential (DP) and collision energy (CE) for individual MRM transitions underwent further optimization. Throughout each elution interval, a predefined set of MRM transitions was monitored based on metabolite retention times.

### 2.4. Qualitative and Quantitative Analysis of Metabolites

Qualitative compound analysis leveraging the curated GB-PLANT database was conducted based on secondary spectral data, during which isotope peaks and recurring adduct signals (K^+^, Na^+^, NH_4_^+^), along with fragment ions from high-molecular-weight compounds, were systematically removed. Metabolite mass spectrometry data across diverse samples were acquired via Analyst 1.6.3 software. Peak areas for all signals were integrated, with relative abundances determined through peak area normalization. Quality control (QC), enforced by an internal standard, ensured analytical consistency; samples exhibiting metabolite RSD > 30% in QC were discarded. Identified compounds were annotated using the KEGG, HMDB, and LipidMaps databases to retrieve classification and pathway details. Based on annotation results, fold-change values were computed and assessed, while statistical significance was evaluated by t-test-derived p-values. OPLS-DA modeling implemented through the R package v3.6.1 ‘ropls’ underwent 200 permutation tests to validate robustness, with Variable Importance in Projection (VIP) values calculated via multi-round cross-validation. A combined approach incorporating the difference multiple, the p-value, and the VIP value of the OPLS-DA model was utilized to screen for differential metabolites whose concentration or abundance exhibits statistically significant changes between distinct biological states, experimental conditions, or sample groups. Screening criteria required a fold change (FC) beyond the threshold of |1| (FC > 1 or FC < −1), combined with a statistically significant *p*-value (<0.05) and Variable Importance in Projection (VIP) >1. Pathway enrichment significance for differential metabolites was assessed via a hypergeometric distribution test applied to KEGG annotations.

### 2.5. RNA-Seq Library Preparation and Sequencing

Total RNA isolation was performed adhering to the Plant RNA Extraction Kit protocol (Sangon Biotech, Shanghai, China). RNA integrity was verified through 1.5% agarose gel electrophoresis, followed by mRNA extraction using poly(A) capture bead-based kits and subsequent synthesis of double-stranded cDNA. The workflow encompassed end-repair, polyadenylation, and adapter ligation. Amplified libraries underwent purification with Hieff NGS™ DNA Selection Beads (Sangon Biotech), prior to sequencing on the Illumina HiSeq™ 2500 platform (Shanghai Bioengineering Co., Shanghai, China).

Transcriptomic data (2019) were provided by Sangon Biotech in Excel format; due to contractual restrictions (Contract No.: MRNA192916QH), raw sequences were inaccessible for public repository deposition. Consequently, analysis focused on 17 unigenes, with FPKM values and GenBank accessions summarized in Table S7.

### 2.6. Transcript Assembly and Analysis

Raw sequencing data (Sequenced Reads) originate from initial Illumina Hiseq™ (San Diego, CA, USA) image files, with QC metrics summarized in a FastQC report containing tabular statistics of raw/clean data and visual assessments. De novo transcript assembly was executed using Trinity (min_kmer_com2 specified; other parameters default), while functional annotations integrated multiple databases (CDD, KOG, COG, NR, NT, PFAM, SwissProt, TrEMBL) via NCBI Blast+. Gene Ontology (GO) categorization leveraged UniProt-aligned protein annotations from SwissProt/TrEMBL.

Preprocessed clean data underwent splicing and assembly generating unigenes, subsequently annotated against bioinformatic repositories. Differential expression analysis identified Unigene expression variations, facilitating functional predictions through GO databases. Ultimately, the AsA biosynthetic pathway was elucidated via GO enrichment analysis. Stage-specific genes modulating AsA metabolism were cross-compared across fruit development phases, selecting significantly altered targets at each stage for experimental validation.

Transcriptomic profiling of three *L. chinense* fruit stages pinpointed phase-dependent differentially expressed genes.

### 2.7. Gene Expression Analysis by RT-qPCR

Gene-specific primers, designed via Primer Premier 5 (Aoke Dingsheng Biotechnology, Beijing, China), were cataloged in Table S8. Total RNA was isolated from *L. chinense* fruits employing the FastPure^®^ (Nanjing, China) Plant RNA Isolation Kit (effective for polysaccharide/polyphenol-rich samples), with integrity verified through 1.2% agarose gel electrophoresis and concentration quantified by BioSpecnano spectrophotometer (Shimadzu, Kyoto, Japan). Synthesized first-strand cDNA utilized Hiscript^®^ RT-qPCR Supermax (Vazyme Biotech, Nanjing, China), with products preserved at −20 °C.

RT-qPCR (20 μL total volume) was executed using ChamQ Universal SYBR Master Mix (Vazyme Biotech, Nanjing, China) under these conditions:

Reaction system: 10 μL 2× Master Mix, 7.2 μL ddH_2_O, 0.4 μL each primer (Table S8), 2 μL cDNA

Cycling protocol:

• 95 °C × 30 s (initial denaturation)
• 40 cycles: 95 °C × 5 s → 60 °C × 30 s
• Melting curve: 95 °C × 15 s → 60 °C × 50 s → 95 °C × 15 s

Normalization relied on *L. chinense* GAPDH (XM_060314892.1), with negative controls included. All reactions, conducted in triplicate using QuantStudio 6 Flex (Applied Biosystems, Invitrogen, Waltham, MA, USA), were instrumentally monitored.

### 2.8. Data Statistics and Analysis

Metabolite principal component analysis (PCA) employed R 3.6.1’s prcomp function, supported by the prcomp package with visualization implemented via factoextra and ggplot2. In PCA score plots, PC1 and PC2 were represented horizontally and vertically, respectively. QuantStudio™ (Thermo Fisher Scientific, Waltham, MA, USA) Real-time PCR software v1.7.2 derived mean values ± SEM from triplicate biological replicates—applied throughout RNA-seq and RT-qPCR datasets. SPSS v20 (IBM) conducted statistical analyses, using one-way ANOVA supplemented with Dunnett’s post hoc test. Correlations between *L. chinense* fruit metabolites and gene expression profiles were assessed through OmicShare tools (https://www.omicshare.com/; accessed on 10 June 2024). Three duplicate samples collected from each fruit, GF, CCF, and RRF, for a total of nine samples, were used for subsequent analysis.

### 2.9. DNA Extraction and High-Throughput Sequencing of Endophytic Bacteria in L. chinense Fruits

Extracted DNA samples served as templates for PCR amplification using universal 16S rDNA V3–V4 primers (F: CADACTCCTACGGGAGGC; R: ATCCTGTTTGMTMCCCVCRC), with sequencing adapters ligated to primer termini. Subsequent purification, quantification, and homogenization generated the sequencing library. Libraries passing quality control underwent Illumina NovaSeq 6000 sequencing. Raw reads underwent primary QC via Trimmomatic v0.33, followed by primer removal using Cutadapt 1.9.1 to yield adapter-free clean reads. The DADA2 pipeline (QIIME2 2020.6) performed denoising, paired-end merging, and chimera filtration, producing final valid non-chimeric sequences.

### 2.10. Correlation Analysis of Endophytic Bacteria with Metabolites

Correlation analysis was conducted between metabolites and endophytic bacteria. The correlation between amino acid metabolites and endophytic bacterial genera was analyzed using the OmicShare tool (https://www.omicshare.cn).

## 3. Results

### 3.1. Analysis of Amino Acid Metabolites in L. chinense Fruits Across Three Developmental Stages

Metabolites of amino acids were obtained from the fruits of *L. chinense* at three different stages of development: GF (green fruit, 16–19 days post-flowering), CCF (color-changing fruit, 22–25 days post-flowering), and RRF (red-ripe fruit, 31–34 days post-flowering) (Figure 1).

**Figure 2:**
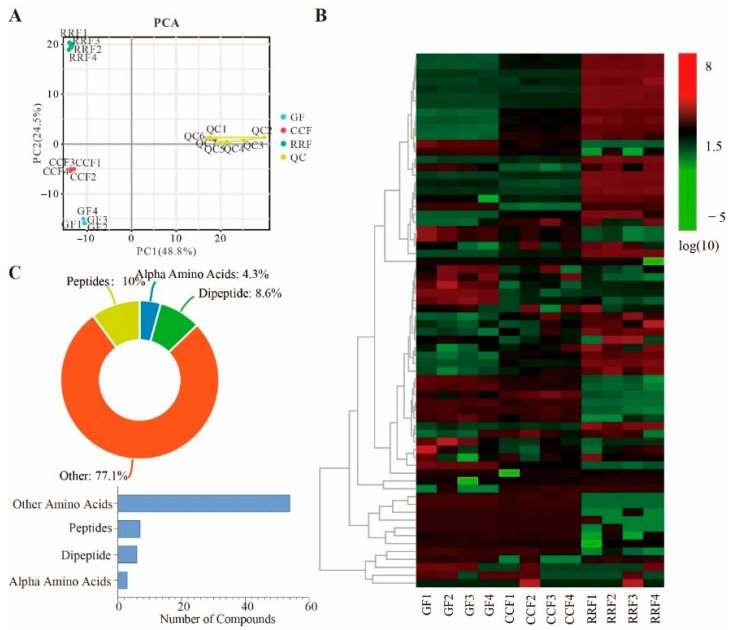
PCA trajectory analysis across phenological stages (GF, CCF, RRF) with QC validation. (**A**) PCA trajectory analysis across phenological stages (GF (green fruit), CCF (color-changing fruit), RRF (red-ripe fruit)), with QC (Quality Control) validation. (**B**) A heat map depicting 600 identified metabolites revealed notable variations in accumulation patterns. The color gradient illustrated the accumulation levels of every metabolite, from low (green) to high (red). (**C**) The categories and proportions of 70 amino acid and related metabolites found in *L. chinense* fruits.

Non-targeted metabolomics of *L. chinense* fruits leveraged Principal Component Analysis (PCA) to evaluate metabolic variability across twelve samples (quadruplicate biological replicates per phenological phase). PCA delineated tight clustering of replicates within identical developmental stages, alongside marked distributional divergences between distinct phenophases (Figure 2A). Consequently, GF, CCF, and RRF samples exhibited differential segregation along PC2, with GF specimens demonstrating pronounced separation from CCF/RRF clusters along PC1.

The comparison and analysis of the number of upregulated and downregulated metabolites in different *L. chinense* fruits were presented in Table S1. The results indicate that amino acid metabolites exhibit the highest counts of both upregulated and downregulated metabolites. This study primarily focuses on the analysis of amino acid metabolites in *L. chinense* fruits, all of which contribute to amino acid synthesis (Table S1). A comparative analysis reveals that there were 27 amino acid metabolites that were upregulated and 24 that were downregulated when comparing the GF and RRF stages. Additionally, between the CCF and RRF stages, 26 amino acid metabolites exhibited upregulation while 16 were downregulated. Furthermore, there were 21 upregulated and 24 downregulated amino acid metabolites observed in the comparison between the GF and CCF stages (Table S1).

In the clustering heatmap, distinct variations in the abundance patterns of 70 amino acid metabolites were observed across various fruit samples (Figure 2B). These metabolites included seven peptides, six dipeptides, three alpha amino acids, and fifty-four other metabolites. Peptides constituted 10% of the total amino acid metabolites, dipeptides 8.6%, alpha amino acids 4.3%, and other metabolites 77.1% (Figure 2C, Table S2).

### 3.2. Analysis of 43 Differential Amino Acid Metabolites Pathway of L. chinense Fruits Across Three Stages

**Figure 3:**
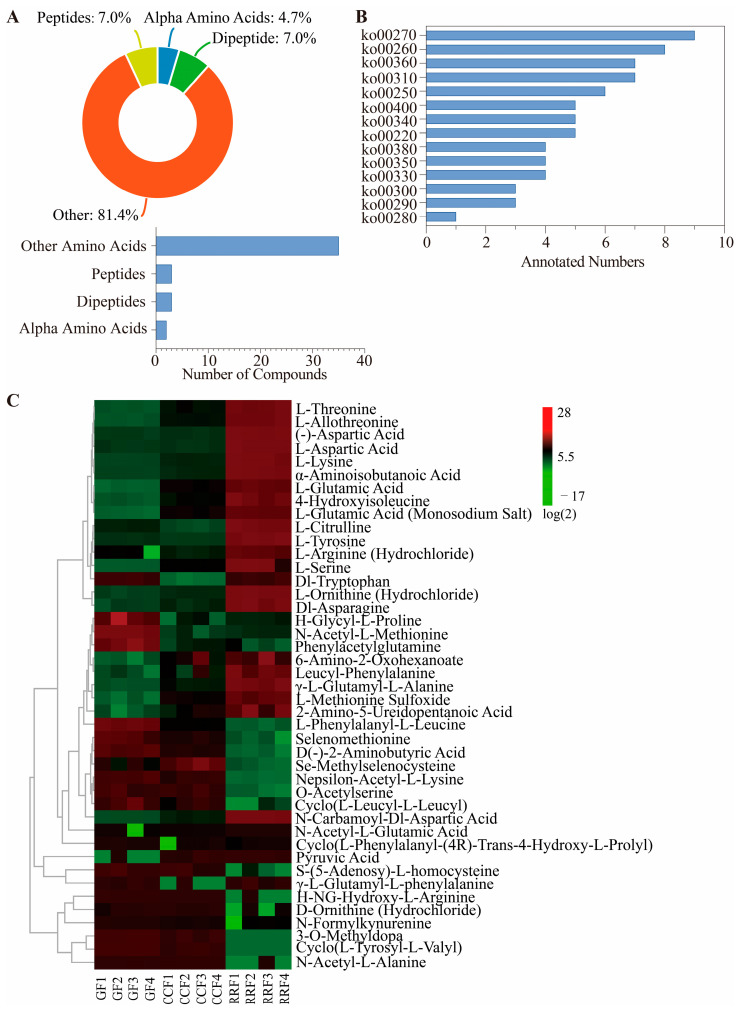
The heatmap and hierarchical cluster illustrating the differentially accumulated metabolites associated with the amino acid metabolites pathway in *L. chinense* fruits across three developmental stages. (**A**) The types and proportions of differential amino acid metabolites annotated in *L. chinense* fruits. (**B**) Classification histogram of 14 amino acid metabolites in *L. chinense* fruit in the KEGG database (ko00220: Arginine biosynthesis; ko00250: Alanine, aspartate and glutamate metabolism; ko00260: Glycine, serine and threonine metabolism; ko00270: Cysteine and methionine metabolism; ko00280: Valine, leucine and isoleucine degradation; ko00290: Valine, leucine and isoleucine biosynthesis; ko00300: Lysine biosynthesis; ko00310: Lysine degradation; ko00330: Arginine and proline metabolism; ko00340: Histidine metabolism; ko00350: Tyrosine metabolism; ko00360: Phenylalanine metabolism; ko00380: Tryptophan metabolism; ko00400: Phenylalanine, tyrosine and tryptophan biosynthesis). (**C**) Stage-specific accumulation dynamics of DAMs, with color gradients indicating relative abundance (green: low, red: high).

To identify amino acid metabolism-associated differentially accumulated metabolites (DAMs) across *L. chinense* fruit phenological stages, screening criteria (fold change [FC] > 2, *p* < 0.05, VIP > 1) were applied. Among seventy characterized amino acid metabolites, forty-three DAMs were classified as follows: three peptides (7.0%), three dipeptides (7.0%), two α-amino acids (4.7%), and thirty-five miscellaneous compounds (81.4%) (Figure 3A; Table S3, Class II). Notably, Pyruvic Acid exhibited a 19.57-fold increase in CCF/GF, while N-Carbamoyl-DL-Aspartic Acid showed an 11.36-fold elevation in RRF/GF. Conversely, L-Phenylalanyl-L-Leucine and 3-O-Methyldopa displayed marked depletion (RRF/GF: 0.19-fold; CCF/GF: 0.00-fold) (Table S3).

A total of 14 categories of amino acid metabolites were successfully matched with the KEGG database (Figure 3B, Table S4). Among these categories, nine metabolites were identified as pertaining to cysteine and methionine metabolism (ko00270), eight metabolites were linked to glycine, serine, and threonine metabolism (ko00260), seven metabolites were associated with lysine degradation (ko00310), and seven metabolites were related to phenylalanine metabolism (ko003609).

The clustering heatmap revealed notable differences in the abundance patterns of 43 differential amino acid metabolites among various fruit samples (see Figure 3C). H-Glycyl-L-proline, N-Acetyl-L-Methionine, phenylacetylglutamine, and L-Methionine Sulfoxide showed high accumulation in GF, while they were low in CCF and RRF. Se-Methylselenocysteine was found to accumulate significantly in CCF, while its levels were low in GF and RRF. Conversely, L-Threonine, L-Allothreonine, (-)-Aspartic Acid, L-Aspartic Acid, L-Lysine, α-Aminoisobutanoic acid, L-Citrulline, L-Tyrosine, L-Ornithine (Hydrochloride), Dl-Asparagine, and N-Carbamoyl-Dl-Aspartic Acid exhibited high accumulation in RRF, while their levels were low in GF and CCF. The findings suggest that the 43 amino acid metabolites with differential levels exhibited unique patterns of accumulation and reduction.

### 3.3. Validation of the Differentially Expressed Genes in AminoAacid Metabolism of L. chinense Fruits

**Figure 4:**
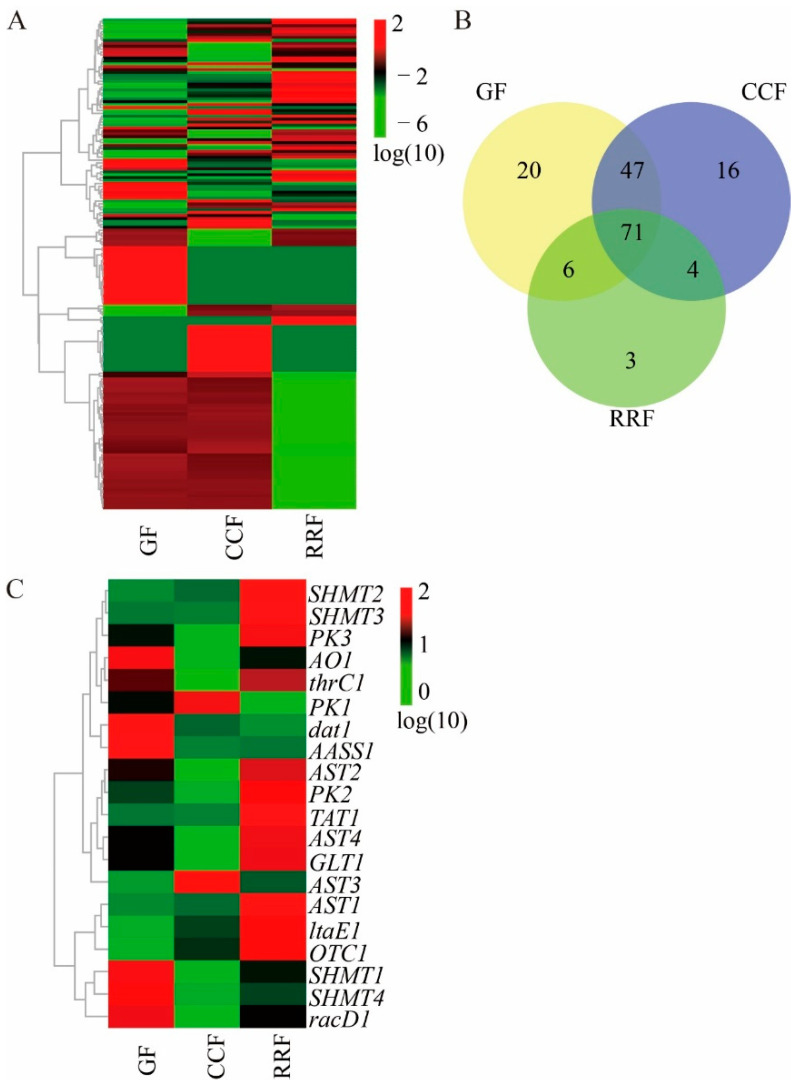
One hundred and seventy amino acid-related gene heatmap, Venn map, and seventeen differentially expressed amino acid-related gene heatmap. (**A**) Transcriptomic heatmap of 170 amino acid biosynthesis-related genes across three phenophases, with color gradients reflecting expression dynamics (green→red: low→high). (**B**) Venn diagram of amino acid biosynthesis-related genes via OmicShare tools (https://www.omicshare.com/; accessed on 3 April 2025). (**C**) Heatmap visualization of 17 differentially expressed genes (DEGs) linked to amino acid metabolic pathways, indicating stage-specific transcript abundance (green→red).

In this research, a total of 170 genes related to the production of amino acids and their derivatives in the fruits of *L. chinense* were depicted in heat maps for three different developmental phases (Figure 4A, Table S5). The findings revealed that most of these genes exhibited expression levels during the first two phases of *L. chinense* fruit development, which was followed by a modest reduction in gene expression during the RRF phase (Figure 4A). Interestingly, of the 170 genes, 71 were common across all three categories (Figure 4B). The uniquely identified genes comprised twenty in GF, sixteen in CCF, and three in RRF (Figure 4B).

The presence of twenty transcripts that code for twelve crucial enzyme-related genes involved in amino acid metabolism was established (see Table S6). Figure 4C illustrates that three enzymes were encoded by multiple unigenes: AST (4), PK (3), and SHMT (4). Conversely, nine essential enzymes were linked to a single unigene: AASS (1), AO (1), dat (1), GLT (1), ltaE (1), OTC (1), racD (1), TAT (1), and thrC (1) (refer to Table S6). A cluster analysis conducted on all differentially expressed genes related to amino acid metabolites across various developmental stages indicated notable differences in gene expression throughout fruit development (see Figure 4C, Table S7). The transcript expressions of *AO1*, *dat1*, *AASS1*, *SHMT1*, *SHMT4,* and *racD1* were elevated in GF, whereas those were reduced in CCF (Figure 4C). The expressions of *PK1* and *AST3* were elevated in CCF, while they were lower in GF and RRF (Figure 4C). The expressions of *SHMT2*, *SHMT3*, *PK3*, *thrC1*, *AST2*, *PK2*, *TAT1*, *AST4*, *GLT1*, *AST1*, and *OTC1* were elevated in RRF (Figure 4C).

**Figure 5:**
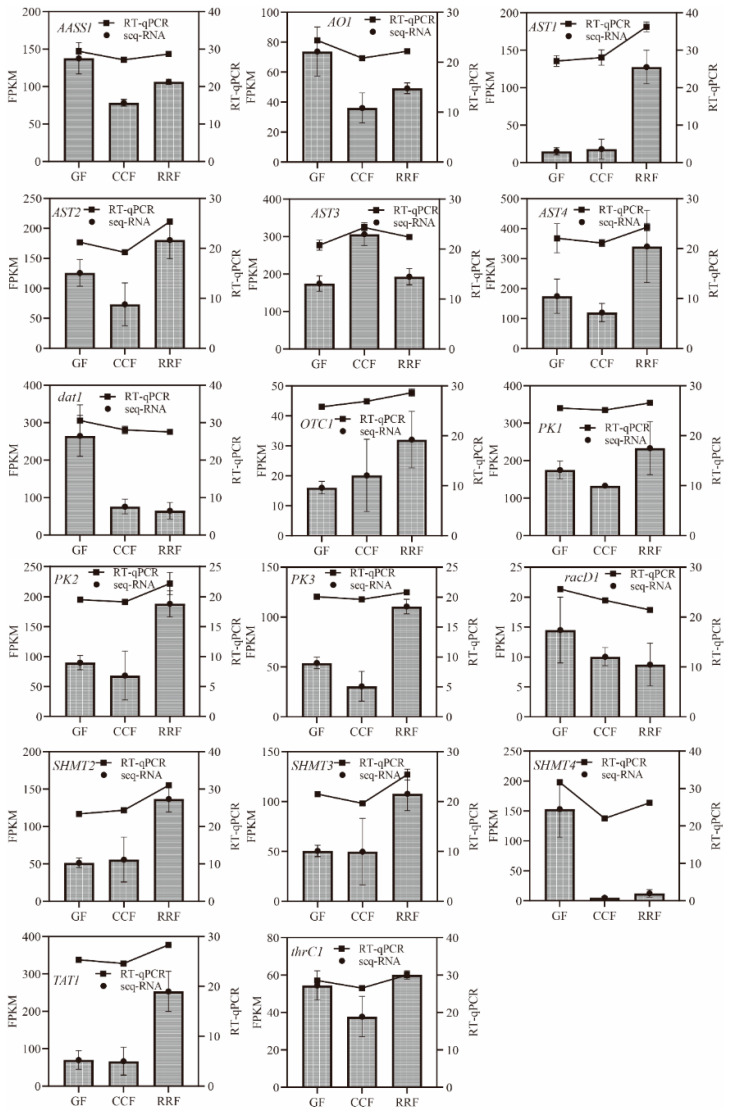
Integrated profiling of amino acid metabolism-associated genes. Transcriptomic (RNA-seq, bars) and validation (RT-qPCR, line) dynamics across *L. chinense* phenophases (GF/CCF/RRF). The column chart displayed the FPKM values for each gene, while the line chart illustrated the relative gene expression by RT-qPCR method. Relative gene expression was calculated using the 2^−ΔΔct^ method. Vertical bars represented means ± SD (*n* = 3) from three replicates. This image was produced using Prism 9.0 software.

To authenticate RNA-seq data reliability, eighteen amino acid metabolism-associated genes were subjected to RT-qPCR validation (Table S8). Normalization of target gene expression levels leveraged the housekeeping gene GAPDH as an endogenous reference. Consistent correlation between RT-qPCR results and transcriptomic profiles was observed across all tested genes, thereby corroborating the robustness of transcriptome data. Within the context of the amino acid metabolic pathway, RT-qPCR analysis revealed that the relative expressions of *dat1* and *racD1* exhibited a downward trend, while *AST1*, *OTC1*, and *SHMT2* demonstrated an upward trend (refer to Figure 5). *AASS1*, *AO1*, *AST2*, *AST4*, *PK1*, *PK2*, *PK3*, *SHMT3*, *SHMT4*, *TAT1*, and *thrC1* displayed an initial decline followed by a rise, in contrast to *AST3*, which showed an initial increase followed by a decrease (see Figure 5).

### 3.4. Analysis of Alpha Diversity of Endophytic Bacteria in L. chinense Fruits at Different Developmental Stages

The α-diversity of endophytic bacterial communities was assessed via Shannon and Chao1 indices. Notably, RRF specimens exhibited reduced ACE, Shannon, and Chao1 values compared to GF/CCF cohorts, whereas Simpson indices remained statistically invariant across all groups (Table 1). As the *L. chinense* fruits developed, the ACE, Chao1, and Shannon indices of endophytic bacteria gradually decreased (Table 1), with particularly pronounced reductions observed in these indices from CCF to RRF.

**Figure 6:**
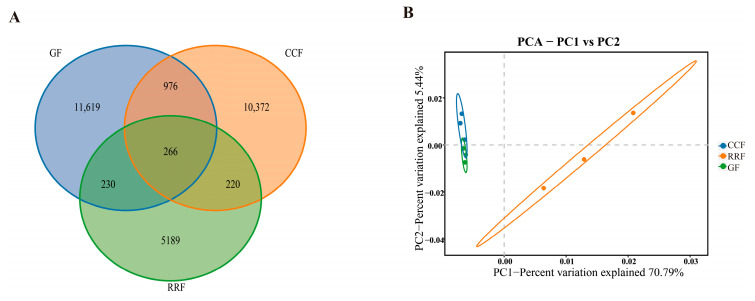
The Venn diagram and PCA analysis of OTUs of endophytic bacteria in *L. chinense* fruits at three developmental stages. (**A**) The Venn diagrams of OTU numbers from endophytic bacteria at different fruits. (**B**) The PCA analysis of endophytic bacteria at different fruit stages.

Following the clustering of the samples, a Venn diagram was created. As illustrated in Figure 6A, variations in the number of endogenous OTUs across different groups were evident. The GF group exhibited the highest number of endophytic bacterial OTUs, followed by CCF, while the RRF group had the lowest number (Figure 6A).

PCA-driven sample classification accentuated inter-sample biodiversity disparities, with microbial community similarity reflected by spatial proximity within the ordination plot. The endophytic bacteria OTUs in the GF group were relatively close to those in the CCF group along the PC1 direction (Figure 6B).

### 3.5. Analysis of Beta Diversity of L. chinense Fruits at Different Development Stages

**Figure 7:**
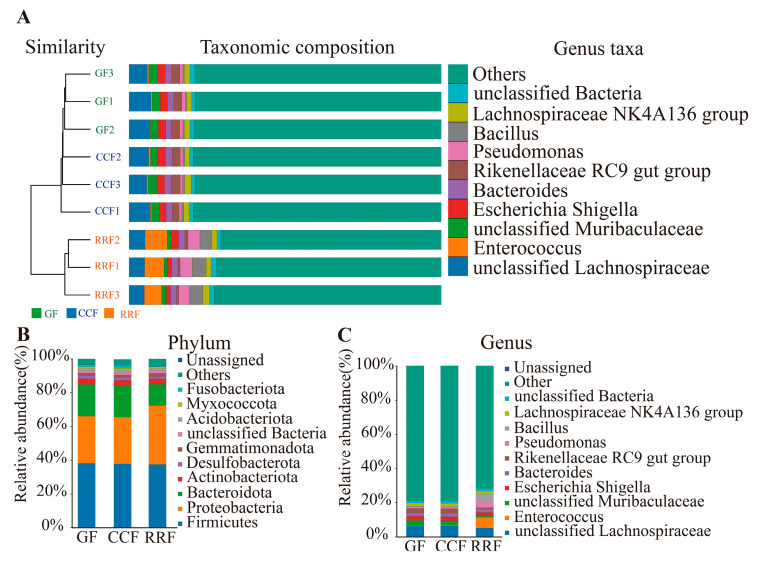
(**A**) Endophytic β analysis of endophytic bacteria in *L. chinense* fruits at three developmental stages. (**B**) Composition and relative abundance of endophytic bacteria at different groups at the phylum level. (**C**) Composition and relative abundance of endophytic bacteria at different groups at the genus level.

The combined UPGMA clustering tree and species composition histogram illustrate the relationships among endophytic bacteria in *L. chinense* fruit across different developmental stages. A sample clustering tree (Figure 7A) was constructed using the weighted_UniFrac distance algorithm, revealing the similarity between sample replicates. The relative abundance of species was analyzed at the genus level. In the UPGMA clustering tree for endophytic bacteria in *L. chinense* fruit across three developmental stages, fruits at the same stage were grouped into a single branch. Notably, in the clustering of GF and CCF, the top five genera of endophytic bacteria with the highest abundance were *unclassified Lachnospiraceae*, *unclassified Muribaculaceae*, *Escherichia Shigella*, *Bacteroides*, and *Rikenellaceae rc9 gut group*, indicating that the endophytic bacterial composition of *L. chinense* fruit remains similar during the GF and CCF periods. In RRF clustering, the top five genera of endophytic bacteria with the highest abundance were *unclassified Lachnospiraceae*, *Enterococcus*, *Escherichia Shigella*, *Pseudomonas*, and *Bacillus*.

### 3.6. Classification of Phyla and Genera of Endophytic Bacteria in L. chinense Fruits

To delineate endophytic compositional variation in *L. chinense* fruits across distinct epiphytic patterns, statistical profiling of annotated phyla and genera was conducted (Figure 7B). Ten predominant bacterial phyla, ranked by abundance, comprised Firmicutes, Proteobacteria, Bacteroidota, Actinobacteriota, Desulfobacterota, Gemmatimonadota, unclassified Bacteria, Acidobacteriota, Myxococcota, and Fusobacteriota. Among these, Firmicutes, Proteobacteria, and Bacteroidota collectively dominated, representing >80% of the total bacterial abundance. Significantly, Proteobacteria exhibited higher prevalence in RRF relative to GF and CCF cohorts.

At the genus level, the relative abundance distribution of the top 10 bacterial genera across all groups was illustrated in Figure 7C. The dominant endophytic genera primarily included *unclassified Lachnospiraceae*, *Enterococcus*, *unclassified Muribaculaceae*, *Escherichia Shigella*, *Bacteroides*, *Rikenellaceae RC9 gut group*, *Pseudomonas*, *Bacillus*, *Lachnospiraceae NK4A136 group*, and *unclassified Bacteria*. Notably, the relative abundance of Enterococcus in the RRF group was higher than that observed in the GF and CCF groups.

### 3.7. Analysis of Dominant Endophytic Bacteria in L. chinense Fruits at Different Developmental Stages

**Figure 8:**
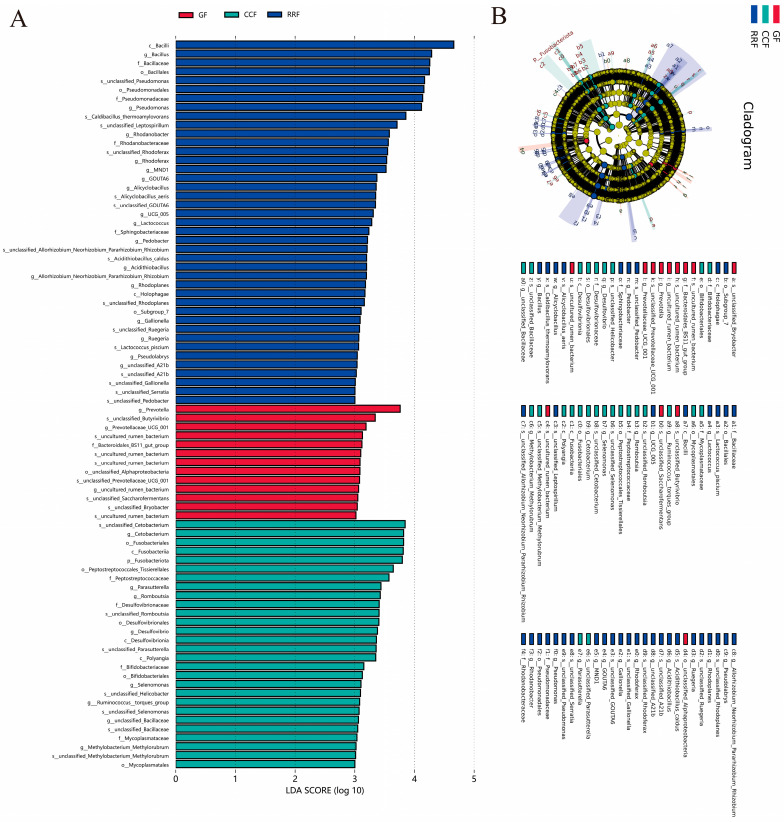
LEfSe and cladogram analysis of endophytic bacterial communities in *L. chinense* fruits at three developmental stages. (**A**) LEfSe analysis of endophytic bacteria in *L. chinense* fruits at GF, CCF, and RRF stages. (**B**) Cladogram analysis of endophytic bacteria in *L. chinense* fruits at GF, CCF, and RRF stages.

LEfSe-based profiling (Figure 8A) identified statistically significant biomarkers of endophytic bacterial communities across *L. chinense* developmental stages, revealing inter-group abundance disparities. The absolute LDA scores quantify differential species effect sizes, with higher values indicating greater taxonomic discriminative power. In the GF group, the significantly enriched taxa include the genera *Prevotell*, *Prevotellaceae UCG 001*, *uncultured rumen bacteria*, and the family Bacteroidales BS11 gut group. Conversely, in the CCF group, the significantly enriched taxa consist of the genera *Cetobacterium*, *Parasutterella*, *Romboutsia*, *Desulfovibrio*, *Selenomonas*, *Ruminococcus torques_group*, and *Methylobacterium Methylorubrum*. The families Peptostreptococcaceae, Desulfovibrionaceae, Bifidobacteriaceae, and Mycoplasmataceae were identified in RRF. Additionally, the significantly enriched taxa included genera *Bacillus*, *Pseudomonas*, *Rhodanobacter*, *Rhodoferax*, *MND1*, *GOUTA6*, *Alicyclobacillus*, *UCG 005*, *Lactococcus*, *Pedobacter*, *Acidithiobacillus*, *Rhodoplanes*, *Gallionella*, *Ruegeria*, *Pseudolabrys*, *unclassified A21b*, along with the families Bacillaceae, Pseudomonadaceae, Rhodanobacteraceae, and Sphingobacteriaceae.

**Figure 9:**
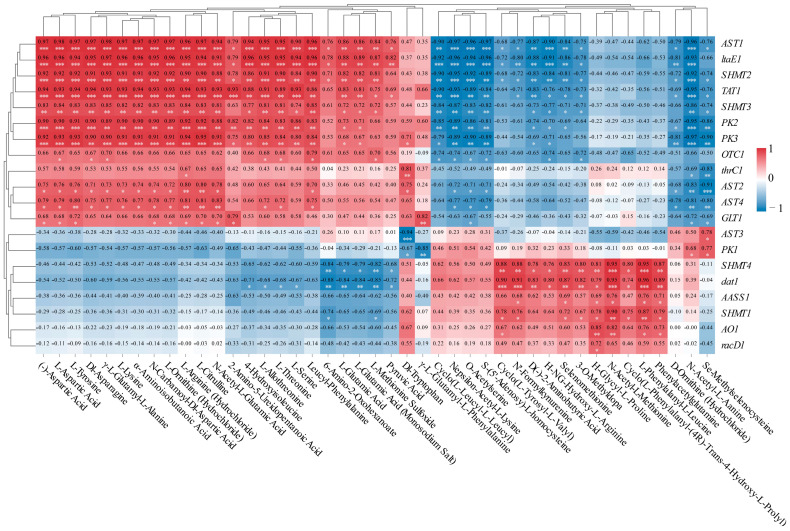
Integrated correlation profiling. Associations between *L. chinense* fruit amino acid metabolism-associated 20 unigenes and 43 metabolites (Red: positive correlation; Blue: negative correlation). Significance thresholds: *p* < 0.05 (*), *p* < 0.01 (**), *p* < 0.001 (***).

The results (Figure 9) indicated that with the development of *L. chinense* fruits, there was a significant increase in diverse endophytic bacteria present in the fruits.

### 3.8. Joint Analysis of Differential Amino Acid Metabolites and Their Related Genes

**Figure 10:**
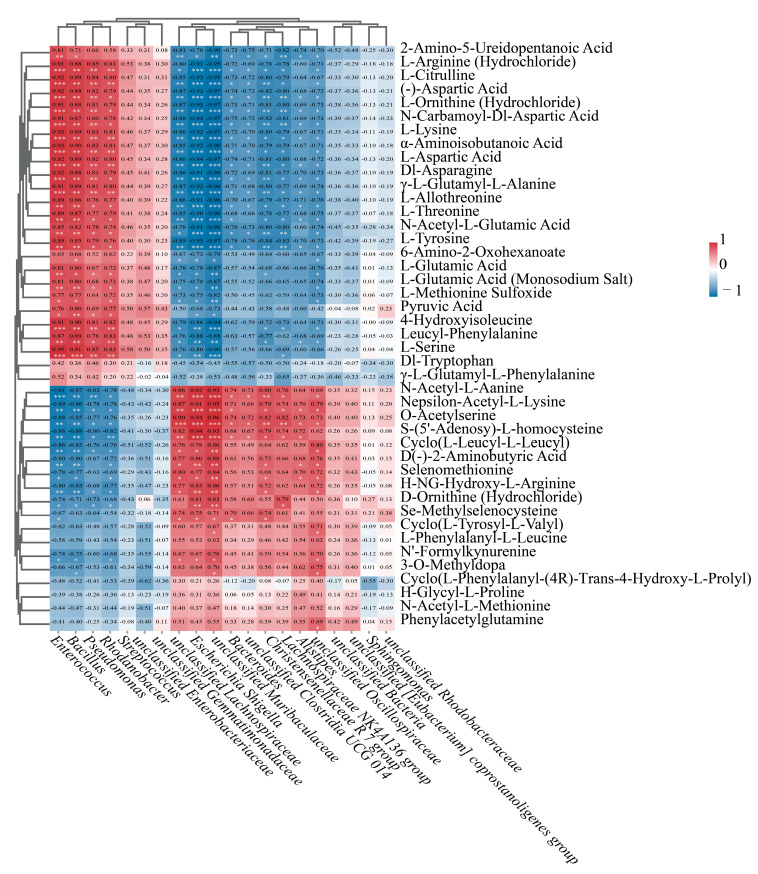
Endophytic bacteria–differential amino acid metabolite correlation heatmap (Red: positive correlation; Blue: negative correlation), wherein color intensity correlates positively with association strength. Significance thresholds: *p* < 0.05 (*), *p* < 0.01 (**), *p* < 0.001 (***).

In order to deepen our comprehension of the molecular processes that contribute to the varying levels of amino acids in the fruits of *L. chinense* at different developmental phases, we investigated the relationship between the differential metabolites and the genes associated with the amino acid pathway using transcriptomic and metabolomic data (Figure 10). This study emphasized the correlation among 20 pertinent genes and 43 compounds linked to amino acids in the fruits of *L. chinense* (Figure 10).

As shown in Figure 10, there was a notable relationship detected between the concentrations of amino acid-related compounds and the expression levels of differentially expressed genes. *AST1* exhibited significantly positive correlations with 23 metabolites, while *SHMT2* showed significantly positive correlations with 22 metabolites. Similarly, *TAT1* demonstrated significantly positive correlations with 22 metabolites, and *SHMT3* revealed significantly positive correlations with 20 metabolites. *PK2* and *PK3* exhibited significantly positive correlations with 20 and 21 metabolites, respectively, as well as with 14 metabolites. *AST4* showed significantly positive correlations with 15 metabolites, and *AST2* exhibited significantly positive correlations with 14 metabolites. *SHMT4* and *dat1* demonstrated significantly positive correlations with the same 10 metabolites. *GLT1* showed significantly positive correlations with 10 metabolites. Finally, *SHMT1* exhibited significantly positive correlations with nine metabolites (Figure 10).

In our study, the gene expressions of *AST1* and *ltaE1* exhibited significantly positive correlations with 23 compounds, with relative indices ranging from 0.76 to 0.98 (*p* < 0.05, *p* < 0.01, *p* < 0.001). Similarly, *SHMT2* and *TAT1* demonstrated significantly positive correlations with 22 distinct compounds, showing relative indices between 0.69 and 0.94 (*p* < 0.05, *p* < 0.01, *p* < 0.001). Furthermore, *SHMT3* displayed significantly positive correlations with 20 different compounds, with relative indices from 0.72 to 0.85 (*p* < 0.05, *p* < 0.01). Consequently, *AST1*, *ltaE1*, *TAT1*, *SHMT2,* and *SHMT3* were identified as key regulatory genes involved in the synthesis of amino acids. Notably, the majority of amino acids that exhibited positive correlations with these five genes were polar amino acids, including L-Aspartic Acid, L-Tyrosine, L-Lysine, L-Threonine, L-Serine, and L-Glutamic Acid. L-Citrulline was identified as a non-essential amino acid, which were amino acids that can be synthesized by the human body from metabolic intermediates. Among these, the essential amino acids—a group of amino acids that cannot be synthesized by the human body and must be obtained exclusively through dietary sources to maintain normal physiological functions, including protein synthesis, tissue repair, and metabolic regulation—were L-Lysine and L-Threonine. These results suggest that the expression of the *AST1*, *ltaE1*, *TAT1*, *SHMT2*, and *SHMT3* genes was positively associated with the synthesis of these seven amino acids.

### 3.9. Joint Analysis of Differential Amino Acid Metabolites and Dominant Endophytic Bacteria

Elucidating the relationship between the fruit endophytic bacteria of *L. chinense* and their metabolites at various developmental stages was crucial for understanding these endophytic bacteria.

This research investigated the relationship between metabolites and microorganisms, focusing on their relative abundance and content. We assessed the correlations among the 20 most abundant genera of endophytic bacteria and 43 metabolites that play a role in the differential synthesis of amino acids in *L. chinense* fruits. Our results indicated that 13 out of the 20 most prevalent genera of endophytic bacteria in *L. chinense* fruits demonstrated highly significant correlations with the differential metabolites of amino acids (*p* < 0.05, *p* < 0.01, *p* < 0.001, Figure 10).

Among the various endophytic bacteria, the four genera *Enterococcus*, *Bacillus*, *Pseudomonas*, and *Rhodanobacter* showed a strong positive association with twenty distinct differential amino acid metabolites, while they displayed a noteworthy negative correlation with seven unique differential amino acid metabolites (*p* < 0.05, *p* < 0.01, *p* < 0.001, Figure 10). Furthermore, the genera *unclassified Lachnospiraceae*, *Escherichia Shigella*, and *unclassified Muribaculaceae* showed a significant positive correlation with eight identical differential amino acid metabolites and a significant negative correlation with twenty-two identical differential amino acid metabolites (*p* < 0.05, *p* < 0.01, *p* < 0.001, Figure 10).

The non-essential amino acid L-Citrulline, along with the polar amino acids L-Aspartic Acid, L-Tyrosine, L-Lysine, L-Threonine, L-Serine, and L-Glutamic Acid, exhibited a positive correlation with the four genera *Enterococcus*, *Bacillus*, *Pseudomonas*, and *Rhodanobacter*.

These findings suggest that the relative abundance of the genera *Enterococcus*, *Bacillus*, *Pseudomonas*, and *Rhodanobacter* was significantly correlated with the relative content of these seven amino acids.

## 4. Discussion

Lu et al. [27] analyzed the composition and content of amino acids in jujube (*Ziziphus jujuba* Mill.) fruits at four different ripening stages, identifying a total of 26 free amino acids, whose overall content diminished progressively as the fruit matured. Li et al. [28] observed that the concentrations of organic acids and amino acids in developing apple (*Malus domestica* L. Borkh.) fruit declined, with the exception of proline and methionine. The levels of free amino acids were primarily constrained by a decreased availability of precursors derived from glycolysis and the TCA cycle [29]. Glycine betaine (GB) has been found to mitigate chilling injury in peach (*Prunus persica* Batsch.) fruit stored under cold conditions post-harvest, and it has been demonstrated to enhance arginine metabolism in peaches, consequently elevating proline levels [29]. Wang et al. [30] investigated the sensory impacts of varying concentrations of cysteine (Cys)—specifically 0%, 0.01%, 0.05%, and 0.10%—on wolfberry (*Lycium Barbarum* L.) fruits preserved at 4 °C with a relative humidity (RH) of 90% over a span of 10 days. Their research revealed that the application of 0.05% Cys significantly increased the levels of proline (Pro) and taurine (Tau), while not notably affecting the amounts of cysteine, glutamic acid (Glu), or gamma-aminobutyric acid (GABA). Marta Vazquez-Vilar et al. [31] successfully created an herbicide-tolerant variant of the commonly expressed acetolactate synthase (mSlALS) gene in tomatoes by incorporating all relevant coding and regulatory DNA components from the tomato genome. This modified tomato exhibited significantly elevated concentrations of leucine (twenty-one times above wild-type levels), valine (nine times above), and isoleucine (three times above) [31].

In *Rubus chingii* (*R. chingii*), leucine, lysine, and phenylalanine emerged as the most abundant amino acids, highlighting their significant presence throughout the growth cycle. The proportion of essential amino acids, relative to the total amino acid content in *R. chingii,* exhibited a notable trajectory of change throughout its developmental stages [32].

*L. chinense* fruits at the RRF stage demonstrated a greater accumulation of amino acid metabolites compared to other stages. We identified 43 differential amino acid metabolites, 21 of which exhibited an increase in content (log2(FC) > 1) during the CCF and RRF stages of *L. chinense* fruit compared to the GF stage (Table S3). For instance, N-Carbamoyl-Dl-Aspartic Acid, α-Aminoisobutanoic acid, and γ-L-Glutamyl-L-phenylalanine showed significant increases in the RRF stage, with increases of 11.36 times, 7.07 times, and 7.42 times, respectively, compared to those in the GF stage (Table S3).

Yan et al. [33] found that red LED irradiation induced the expression of genes encoding *AST* in harvested broccoli, maintained amino acid content, and promoted amino acid anabolism, resulting in a delay in broccoli senescence. When Sweet orange ‘Newhall’ (*C. sinensis*) was grafted onto two different rootstocks, *Poncirus trifoliata* (CT) and *Citrus. junos* *Siebold ex Tanaka* (CJ), the expression levels of both the *AST* and *PK* genes in the peel were altered [34]. In citrus peel [35], the upregulation of *SHMT* gene expression may be associated with oleocellosis and lead to the accumulation of glutamate, valine, glycine, and threonine in citrus fruits. Xin et al. [36] proposed that *TAT* plays an important role in 1-deoxynojirimycin (DNJ) biosynthesis in mulberry (*Morus alba* L.) seeds.

In our study, *AST1*, *ltaE1*, *TAT1*, *SHMT2,* and *SHMT3* were identified as key regulatory genes involved in the synthesis of amino acids, exhibiting a positive correlation with L-Aspartic Acid, L-Tyrosine, L-Lysine, L-Threonine, L-Serine, L-Glutamic Acid, and L-Citrulline.

Numerous studies have demonstrated that endophytic bacteria enhance drought resistance in host plants by regulating the concentration of osmoregulatory substances and the activity of the antioxidant system [37]. Additionally, these bacteria increase the activity of peroxidases, such as ascorbate peroxidase (APX) and antioxidant oxidase (AO), thereby fortifying the plant’s antioxidant system [37,38]. Furthermore, certain endophytic bacteria can elevate the concentrations of ascorbic acid and glutathione in plants exposed to drought conditions [39].

Among the endophytic bacteria that exhibited a significant correlation with differential metabolites of amino acids, the genera *Enterococcus* and *Bacillus* emerged as the most frequently utilized microorganisms as probiotics in various non-dairy products [40]. Moreover, prebiotic components such as polyphenols [41] could have a beneficial impact on gut health. Additionally, the *Bacillus* genus, which was naturally found on fruit surfaces, can be employed in fermentation methods [42]. In fruits subjected to four pretreatments with *Pseudomonas fluorescens* ZX, the levels of hesperidin, sinensetin, nobiletin, synephrine, and pectin increased by approximately 26.0%, 31.3%, 44.8%, 19.7%, and 23.1%, respectively, compared to the untreated control group. Overall, these findings suggest that utilizing *P. fluorescens* ZX as a biostimulant through preharvest application represents an effective, cost-efficient, and environmentally friendly strategy for enhancing citrus crop production [43]. Furthermore, inducing blackberry (*Rubus* sp.) plants through root application of *P. fluorescens* N21.4 resulted in enhanced expression of specific flavonoid biosynthetic genes and a concomitant increase in the levels of certain flavonoids within the fruits. This phenomenon may be linked to the ability of plant growth-promoting rhizobacteria (PGPR) to stimulate flavonoid synthesis as part of an induced systemic response (ISR), highlighting the significant role this pathway plays in plant defense, which results in elevated concentrations of flavonoids in the fruit [44]. In soybean cultures, compared with uninoculated soybeans, *Bacillus subtilis* increased the amounts of leucine and phenylalanine; *B. velezensis* increased the amounts of leucine, phenylalanine, and tyrosine; and *B. licheniformis* increased the amounts of alanine, glutamic acid, tyrosine, and ornithine and dramatically decreased the amount of arginine [45]. *Enterococci* provide fermentable amino acids, including leucine and ornithine, which increase *Clostridioides* difficile fitness in the antibiotic-perturbed gut [46].

In our study, the endophytic bacterial genera *Enterococcus*, *Bacillus*, and *Pseudomonas* exhibited significant positive correlations with numerous differential amino acid metabolites. The function prediction of *Bacillus* included global and overview maps, such as amino acid metabolism. These endophytic bacteria may be closely associated with the metabolism of amino acids in *L. chinense* fruit.

## 5. Conclusions

This article analyzes the three developmental stages of *L. chinense* fruit—green fruit (GF), color-changing fruit (CCF), and red-ripe fruit (RRF)—through the lenses of metabolomics, transcriptomics, and microbiology. A total of 43 differential amino acid metabolites were identified in *L. chinense* fruits across these three developmental stages. Among the genes related to amino acid synthesis, six were found to be involved in the arginine biosynthesis pathway (ko00220), six in glycine, serine, and threonine metabolism (ko00260), and five in alanine, aspartate, and glutamate metabolism (ko00250). Key regulatory genes associated with amino acid synthesis, specifically *AST1*, *ltaE1*, *TAT1*, *SHMT2*, and *SHMT3,* were identified. Furthermore, *Bacillus*, *Enterococcus*, *Rhodanobacter*, and *Pseudomonas* were recognized as the primary endophytic bacterial genera in *L. chinense* fruit. These genera exhibited a positive correlation with seven amino acids (L-Aspartic Acid, L-Tyrosine, L-Lysine, L-Threonine, L-Serine, L-Glutamic Acid, and L-Citrulline), influencing their metabolism within the fruit. These findings provide insights into the relationship between amino acid synthesis and endophytic bacteria in *L. chinense* fruit.

## Figures and Tables

**Table 1:** Alpha diversity indices of endophytic bacteria in *L. chinense* fruits at three developmental stages.

	Raw Reads	Clean Reads	Clean Bases	Q20 Bases Ratio (%)	Q30 Bases Ratio (%)	GC Bases Ratio (%)
GF	46,291,638 ± 864,646	45,160,076 ± 754,771	6.27G ± 0.23G	99.02% ± 0.01%	96.05% ± 0.04%	44.28% ± 0.10%
CCF	47,244,229 ± 1,286,887	45,764,233 ± 1,321,919	6.27G ± 0.23G	98.83% ± 0.02%	95.49% ± 0.06%	44.77% ± 0.96%
RRF	44,352,115 ± 1,435,157	44,720,502 ± 44,352,115	5.94G ± 0.20G	98.86% ± 0.03%	95.64% ± 0.07%	43.34% ± 0.16%

## Data Availability

In our study, GenBank Numbers of 17 unigenes were in Table S8. Endophytic bacteria data are available at https://dataview.ncbi.nlm.nih.gov/object/PRJNA1210065?reviewer=smg856b1kvspn80gmr2gqe3ev0 (accessed on 26 June 2025).

## Supplementary Materials

The following supporting information can be downloaded at: https://www.mdpi.com/article/10.3390/genes16070836/s1, Table S1: The number of differentially metabolites in *L. chinense *fruits among three stages; Table S2: 70 Identified Amino acids in *L. chinense* fruits; Table S3: 43 differential metabolites of Amino acids in *L. chinense *fruits among three stages; Table S4: 14 Amino Acid Metabolites Categories of *L. chinense* Fruit in KEGG database; Table S5: 170 genes associated with the synthesis of amino acids in *L. chinense* fruit; Table S6: 20 Unigenes in differential Amino acid metabolism of *L. chinense *fruits; Table S7: FPKM of 20 Unigenes; Table S8: Primers and Sequences For RT-qPCR.

## Abbreviations

The following abbreviations are used in this manuscript:

AASS	Alpha-aminoadipic semialdehyde synthase
AO	Acetylornithine deacetylase
AST	Aspartate aminotransferase
dat	D-alanine transaminase
GLT	Glutamate synthase
ltaE	Threonine aldolase
OTC	Ornithine carbamoyltransferase
PK	Pyruvate kinase
racD	Aspartate racemase
SHMT	Serine hydroxymethyltransferase
thrC	Threonine synthase
TAT	Tyrosine aminotransferase

## References

[1] Moormann J., Heinemann B., Hildebrandt T.M. (2022). News about Amino Acid Metabolism in Plant–Microbe Interactions. Trends Biochem. Sci..

[2] Glawischnig E. (2007). Camalexin. Phytochemistry.

[3] Fagundes M.A. (2021). Investigation of Methionine and Lysine Derivatives as a Source of Rumen-Protected Amino Acids for Lactating Dairy Cows. Ph.D. Thesis.

[4] Umehara T., Kosono S., Soll D., Tamura K. (2018). Lysine Acetylation Regulates Alanyl-tRNA Synthetase Activity in *Escherichia coli*. Genes.

[5] Yao X., Nie J., Bai R., Sui X. (2020). Amino Acid Transporters in Plants: Identification and Function. Plants.

[6] Kim D.-R., Kwak Y.-S. (2023). Endophytic Streptomyces Population Induced by L-Glutamic Acid Enhances Plant Resilience to Abiotic Stresses in Tomato. Front. Microbiol..

[7] Chitnis V.R., Suryanarayanan T.S., Nataraja K.N., Prasad S.R., Oelmüller R., Shaanker R.U. (2020). Fungal Endophyte-Mediated Crop Improvement: The Way Ahead. Front. Plant Sci..

[8] Tedersoo L., Bahram M., Zobel M. (2020). How Mycorrhizal Associations Drive Plant Population and Community Biology. Science.

[9] Tiwari P., Bajpai M., Singh L.K., Yadav A.N., Bae H., Yadav A.N. (2021). Portraying Fungal Mechanisms in Stress Tolerance: Perspective for Sustainable Agriculture. Recent Trends in Mycological Research: Volume 1: Agricultural and Medical Perspective.

[10] Tiwari P., Kang S., Bae H. (2023). Plant-Endophyte Associations: Rich yet under-Explored Sources of Novel Bioactive Molecules and Applications. Microbiol. Res..

[11] Hettiarachchige I.K., Elkins A.C., Reddy P., Mann R.C., Guthridge K.M., Sawbridge T.I., Forster J.W., Spangenberg G.C. (2019). Genetic Modification of Asexual Epichloë Endophytes with the perA Gene for Peramine Biosynthesis. Mol. Genet. Genomics.

[12] Yang C.-C., Chien J.-Y., Chou Y.-Y., Ciou J.-W., Huang S.-P. (2022). The Effects of *Lycium chinense*, *Cuscuta chinensis*, *Senna tora*, *Ophiopogon japonicus*, and *Dendrobium nobile* Decoction on a Dry Eye Mouse Model. Med. Kaunas Lith..

[13] Teixeira F., Silva A.M., Delerue-Matos C., Rodrigues F. (2023). *Lycium barbarum* Berries (Solanaceae) as Source of Bioactive Compounds for Healthy Purposes: A Review. Int. J. Mol. Sci..

[14] Fu Y.-W., Peng Y.-F., Huang X.-D., Yang Y., Huang L., Xi Y., Hu Z.-F., Lin S., So K.-F., Ren C.-R. (2021). *Lycium barbarum* Polysaccharide-Glycoprotein Preventative Treatment Ameliorates Aversive. Neural Regen. Res..

[15] Wang Y., Wei W., Guo M., Li S., Chai Z., Ma C., Jiang Y., Song L., Yu J. (2021). *Lycium barbarum* Polysaccharide Promotes M2 Polarization of BV2 Microglia Induced by LPS via Inhibiting the TLR4/NF-κB Signaling Pathway. Xi Bao Yu Fen Zi Mian Yi Xue Za Zhi Chin. J. Cell. Mol. Immunol..

[16] Li X., Mo X., Liu T., Shao R., Teopiz K., McIntyre R., So K.-F., Lin K. (2022). Efficacy of *Lycium barbarum* Polysaccharide in Adolescents with Subthreshold Depression: Interim Analysis of a Randomized Controlled Study. Neural Regen. Res..

[17] Liang R., Zhao Q., Zhu Q., He X., Gao M., Wang Y. (2021). *Lycium barbarum* Polysaccharide Protects ARPE-19 Cells against H_2_O_2_-induced Oxidative Stress via the Nrf2/HO-1 Pathway. Mol. Med. Rep..

[18] Pan H., Niu L., Wu Y., Chen L., Zhou X., Zhao Y. (2021). *Lycium barbarum* Polysaccharide Protects Rats and Cardiomyocytes against Ischemia/Reperfusion Injury via Nrf2 Activation through Autophagy Inhibition. Mol. Med. Rep..

[19] Qiao F., Zhang K., Zhou L., Qiu Q.-S., Chen Z., Lu Y., Wang L., Geng G., Xie H. (2022). Analysis of Flavonoid Metabolism during Fruit Development of *Lycium chinense*. J. Plant Physiol..

[20] Yin C., Xie H., Geng G., Li Z., Ma J., Wu X., Qiu Q.-S., Qiao F. (2024). Identification of Key Enzymes and Genes Modulating L-Ascorbic Acid Metabolism During Fruit Development of *Lycium chinense* by Integrating Metabolome, Transcriptome, and Physiological Analysis. Int. J. Mol. Sci..

[21] Ding H., Wang J., Zhang X.-Y., Yin L., Feng T. (2021). *Lycium barbarum* Polysaccharide Antagonizes LPS-Induced Inflammation by Altering the Glycolysis and Differentiation of Macrophages by Triggering the Degradation of PKM2. Biol. Pharm. Bull..

[22] Yin R., Xue J., Tan Y., Fang C., Hu C., Yang Q., Mei X., Qi D. (2021). The Positive Role and Mechanism of Herbal Medicine in Parkinson’s Disease. Oxid. Med. Cell. Longev..

[23] Wu I.-H., Chan S.-M., Lin C.-T. (2020). The Neuroprotective Effect of Submicron and Blended *Lycium barbarum* for Experiment Retinal Ischemia and Reperfusion Injury in Rats. J. Vet. Med. Sci..

[24] Tian X., Liang T., Liu Y., Ding G., Zhang F., Ma Z. (2019). Extraction, Structural Characterization, and Biological Functions of *Lycium barbarum* Polysaccharides: A Review. Biomolecules.

[25] Xu L., Yang L., Xu H., Li Y., Peng F., Qiu W., Tang C. (2024). *Lycium barbarum* Glycopeptide Ameliorates Motor and Visual Deficits in Autoimmune Inflammatory Diseases. Phytomedicine Int. J. Phytother. Phytopharm..

[26] Lakshmanan Y., Wong F.S.Y., So K.-F., Chan H.H.-L. (2024). *Lycium barbarum* Glycopeptide Promotes Neuroprotection in ET-1 Mediated Retinal Ganglion Cell Degeneration. J. Transl. Med..

[27] Lu Y., Bao T., Mo J., Ni J., Chen W. (2021). Research Advances in Bioactive Components and Health Benefits of Jujube (*Ziziphus jujuba* Mill.) Fruit. J. Zhejiang Univ.-Sci. B.

[28] Li M., Li D., Feng F., Zhang S., Ma F., Cheng L. (2016). Proteomic Analysis Reveals Dynamic Regulation of Fruit Development and Sugar and Acid Accumulation in Apple. J. Exp. Bot..

[29] Jia Z., Wang Y., Wang L., Zheng Y., Jin P. (2022). Amino Acid Metabolomic Analysis Involved in Flavor Quality and Cold Tolerance in Peach Fruit Treated with Exogenous Glycine Betaine. Food Res. Int. Ott. Ont..

[30] Wang J., Wei L., Yan L., Zheng H., Liu C., Zheng L. (2022). Effects of Postharvest Cysteine Treatment on Sensory Quality and Contents of Bioactive Compounds in Goji Fruit. Food Chem..

[31] Vazquez-Vilar M., Fernandez-del-Carmen A., Garcia-Carpintero V., Drapal M., Presa S., Ricci D., Diretto G., Rambla J.L., Fernandez-Muñoz R., Espinosa-Ruiz A. (2023). Dually Biofortified Cisgenic Tomatoes with Increased Flavonoids and Branched-chain Amino Acids Content. Plant Biotechnol. J..

[32] Luo Y., Hua Y., Chen S., Qian X., Ruan H., Pan P., Chen H. (2024). Widely Untargeted Metabolomics Profiling Combined with Transcriptome Analysis Provides New Insight into Amino Acid Biosynthesis at Different Developmental Stages of *Rubus chingii* Hu (Chinese Raspberry). J. Med. Food.

[33] Yan Z., Xu D., Yue X., Yuan S., Shi J., Gao L., Wu C., Zuo J., Wang Q. (2023). Whole-Transcriptome RNA Sequencing Reveals Changes in Amino Acid Metabolism Induced in Harvested Broccoli by Red LED Irradiation. Food Res. Int. Ott. Ont..

[34] Xiong B., Li Q., Yao J., Zheng W., Ou Y., He Y., Liao L., Wang X., Deng H., Zhang M. (2023). Transcriptome and UPLC-MS/MS Reveal Mechanisms of Amino Acid Biosynthesis in Sweet Orange “Newhall” after Different Rootstocks Grafting. Front. Plant Sci..

[35] Xie J., Deng B., Wang W., Zhang H. (2023). Changes in Sugar, Organic Acid and Free Amino Acid Levels and the Expression of Genes Involved in the Primary Metabolism of Oleocellosis in Citrus Peels. J. Plant Physiol..

[36] Xin X., Jiang X., Thomas A., Niu B., Zhang M., Xu X., Zhang R., Li H., Gui Z. (2024). Studies on 1-Deoxynojirimycin Biosynthesis in Mulberry (*Morus alba* L.) Seeds through Comparative Transcriptomics. Nat. Prod. Res..

[37] Govindasamy V., George P., Kumar M., Aher L., Raina S.K., Rane J., Annapurna K., Minhas P.S. (2020). Multi-Trait PGP Rhizobacterial Endophytes Alleviate Drought Stress in a Senescent Genotype of Sorghum [*Sorghum bicolor* (L.) Moench]. 3 Biotech.

[38] Husna H., Hussain A., Shah M., Hamayun M., Iqbal A., Qadir M., Asim S., Lee I.-J. (2022). *Stemphylium lycopersici* and *Stemphylium solani* Improved Antioxidant System of Soybean under Chromate Stress. Front. Microbiol..

[39] Cao J.-L., He W.-X., Zou Y.-N., Wu Q.-S. (2023). An Endophytic Fungus, *Piriformospora indica*, Enhances Drought Tolerance of Trifoliate Orange by Modulating the Antioxidant Defense System and Composition of Fatty Acids. Tree Physiol..

[40] Cosme F., Inês A., Vilela A. (2022). Consumer’s Acceptability and Health Consciousness of Probiotic and Prebiotic of Non-Dairy Products. Food Res. Int. Ott. Ont..

[41] Vilela A., Cosme F., Inês A. (2020). Wine and Non-Dairy Fermented Beverages: A Novel Source of pro- and Prebiotics. Fermentation.

[42] Kimura K., Yokoyama S. (2019). Trends in the Application of Bacillus in Fermented Foods. Curr. Opin. Biotechnol..

[43] Wang Z., Chen X., Zhong T., Li B., Yang Q., Du M., Zalán Z., Kan J. (2021). Bioeffector Pseudomonas Fluorescens ZX Elicits Biosynthesis and Accumulation of Functional Ingredients in Citrus Fruit Peel: A Promising Strategy for a More Sustainable Crop. J. Agric. Food Chem..

[44] Garcia-Seco D., Zhang Y., Gutierrez-Mañero F.J., Martin C., Ramos-Solano B. (2015). Application of Pseudomonas Fluorescens to Blackberry under Field Conditions Improves Fruit Quality by Modifying Flavonoid Metabolism. PLoS ONE.

[45] Seo S., Jeong D.-W., Sul S., Lee J.-H. (2024). Specificity of Amino Acid Profiles Produced in Soybean Fermentations by Three *Bacillus* spp.. J. Microbiol. Biotechnol..

[46] Smith A.B., Jenior M.L., Keenan O., Hart J.L., Specker J., Abbas A., Rangel P.C., Di C., Green J., Bustin K.A. (2022). Enterococci Enhance Clostridioides Difficile Pathogenesis. Nature.

