# Supplementary material for: Diversity and Correlation Analysis of Differential Amino Acid Metabolites and Dominant Endophytic Bacteria in Lycium chinense Fruits at Different Stages
Source: Genes (Basel). 2025 Jul 18;16(7):836. doi: 10.3390/genes16070836 (PMC12294258; doi:10.3390/genes16070836)
Supplement: Supplementary file 1 [file genes-16-00836-s001.zip › genes-3708486-supplementary.pdf]

Table S1. The number of differentially metabolites in *L. chinense* fruits among three stages

group	GF vs RRF		GF vs CCF		CCF vs RRF	
	Up-regulated	Down-regulated	Up-regulated	Down-regulated	Up-regulated	Down-regulated
Alkaloids	13	24	9	26	13	18
Amino acids	27	24	21	24	26	16
Coumarins	2	4	2	4	4	3
Flavonoids	4	17	7	13	7	17
Ketones, Aldehydes, Acids	6	7	4	6	4	5
Lignans	1	4	0	4	0	2
Lipid	5	9	7	9	3	7
Nucleosides	0	1	0	0	0	1
Nucleotides	2	5	1	5	3	4
Organic acid	17	18	9	19	19	19
Phenylpropanoids	0	4	2	3	0	2
Polyphenols	9	12	7	14	9	12
Quinones	0	1	0	2	0	0
Sugars and alcohols	12	15	25	7	14	16
Steroids	1	2	1	2	1	3
Terpenoids	11	20	9	17	11	17
Vitamins	1	2	0	3	2	2
Xanthones	0	2	0	0	0	0
Others	10	19	8	13	12	17
Total	121	190	112	171	128	161

Table S2. 70 Identified Amino acids in L. chinense fruits

	name	Class I	Class II	GF1	GF2	GF3	GF4	CCF1	CCF2	CCF3	CCF4	RRF1	RRF2	RRF3	RRF4
1	Glycylglycine	Amino acids	Peptides	3615021	3634033	3562678	4456231	5283755	5262857	6177468	5005235	5965641	4224565	4140642	6157077
2	Glycyl-L-Leucine	Amino acids	Peptides	86583.08	95842.8	88081.74	82857.69	60001.85	72651.6	86753.81	77603.24	51836.87	145816	149087.2	63863.64
3	γ-L-Glutamyl-L-Alanine	Amino acids	Peptides	102718.5	93616.68	98676.56	98695.69	230878.1	180038.3	187756.9	190897.2	1007544	967609.2	879017.3	1065207
4	Aurantiamide Acetate	Amino acids	Peptides	45349253	42457794	42211777	41735286	35450775	38000033	35896215	33735402	25619398	26291934	26024018	26434553
5	L-Phenylalanyl-L-Leucine	Amino acids	Peptides	172565.9	150977.2	164373.7	145139.4	33557.2	27996.89	31603.75	27623	6247.335	6503.595	4813.548	7003.385
6	L-Phenylalanyl-L-Tryptophan	Amino acids	Peptides	39493.87	48366.24	12975.76	1.14E-05	1.14E-05	7436.458	1.14E-05	1.14E-05	1.14E-05	1.14E-05	1.14E-05	38342.02
7	Pyruvic Acid	Amino acids	Peptides	1.14E-05	19046.98	1.14E-05	1.14E-05	48342.44	29321.99	174323.9	120715.2	185422.2	129115.3	156637.7	100439.5
8	Cyclo(Tyr-Val)	Amino	Dipeptide	314176.4	331394.6	382041.8	340623.7	35314.17	84656.69	141296.3	68899.58	1.14E-05	1.14E-05	1.14E-05	1.14E-05

		acids													
9	N-((-)-Jasmonoyl)-S-Isoleucine	Amino acids	Dipeptide	7281.358	2182.991	676.2454	3145.176	5593.707	1902.486	4762.941	3643.812	9484.515	3594.765	4323.576	4115.398
10	Cyclo(Hpro-Leu)	Amino acids	Dipeptide	302164.1	340710.7	314214	515477.8	519187.2	583923.8	501592.5	404899.4	513629.8	539526.6	505797.9	289690.7
11	Cyclo(Ile-Leu)	Amino acids	Dipeptide	64544859	65984242	63095723	58306609	46429672	46325826	42561710	46239372	63043304	70391094	54824956	71762669
12	Cyclo(Leu-Leu)	Amino acids	Dipeptide	740169.4	875264.1	1199467	747422.5	431570.9	658724.6	766702.5	673443.8	68221.23	70176.95	242095.4	165467.6
13	Cyclo(Phe-Hpro)	Amino acids	Dipeptide	226748	261219.6	167593.4	192810.9	1.14E-05	114995.5	110530.5	163549	27301.36	114568.5	129817.5	134380.9
14	3-(Carboxymethylamino) Propanoic Acid	Amino acids	Alpha Amino Acids	23118416	23518845	22554815	23829338	39954347	39295541	37778109	38662269	49862541	45872210	47553112	42351350
15	Dl-Tryptophan	Amino acids	Alpha Amino Acids	8870580	8933480	9090941	8545633	1894040	1593057	1719564	1795526	8558258	8894143	8666744	9541360
16	N'-Formylkynurenine	Amino acids	Alpha Amino Acids	339718	277390.2	313477.9	308128.1	127446.1	111924.7	154798.6	120253.4	1.14E-05	22708.66	38623.26	13116.81
1	4-Hydroxyisoleucine	Ami	Other	19504	19953	19347	18861	26107	27888	27375	27473	48566	45886	43098	45261

7		no acids		221	717	397	588	768	053	961	195	093	411	213	951
18	Ac-Ala-Oh	Amino acids	Other	126973.1	125960.7	132716.4	122609.7	134766.4	156594.7	160741.6	148903.8	1.14E-05	1.14E-05	25512.78	1.14E-05
19	(-)-Aspartic Acid	Amino acids	Other	10563773	10744553	10732834	10224388	11284616	11269674	11014180	11358501	33496067	33184389	32492572	33189840
20	Dl-Arginine	Amino acids	Other	29076589	29064527	30066918	29944349	24758441	24075372	24354296	25694741	10586692	27103303	9760866	24251126
21	Dl-Asparagine	Amino acids	Other	46584140	52012319	51438359	50837421	58915038	54263449	56663155	60055817	1.21E+08	1.22E+08	1.14E+08	1.2E+08
22	Dl-Tyrosine	Amino acids	Other	1.14E-05	1.14E-05	1.14E-05	1.14E-05	1.14E-05	1.14E-05	1.14E-05	1.14E-05	1.14E-05	1.14E-05	1.14E-05	1.14E-05
23	L-Allothreonine	Amino acids	Other	8325609	8472422	8303407	8684266	12891818	12998829	13166800	13054038	27915207	27923396	27961537	28772499
24	L-Aspartic Acid	Amino acids	Other	15595644	14791809	14730225	15103511	15616000	15218776	15260101	15878517	43007388	41898714	41751322	41861665
25	L-Glutamic Acid	Amino acids	Other	17079446	18216069	17747118	18015285	32106031	32209559	30159896	32096845	47071534	48437535	47058826	46201216

26	L-Glutamic Acid (Monosodium Salt)	Amino acids	Other	15012287	14519085	14383136	13773467	28344432	28952293	27346667	29666892	43806488	43132801	42386320	42758584
27	L-Methionine Sulfoxide	Amino acids	Other	167787.7	150625.2	172186.8	151311.9	265115.9	258113.9	249616.8	251461.5	344937.2	322202.7	338174.6	346248.6
28	L-Ornithine (Hydrochloride)	Amino acids	Other	88120155	80945589	86501667	86204191	98423933	1.02E+08	1.01E+08	1E+08	3E+08	3.04E+08	2.96E+08	2.95E+08
29	L-Serine	Amino acids	Other	2315050	2328657	2362977	2394324	6065187	5958396	6176461	5835672	20432500	20875816	20254034	8505247
30	L-Threonine	Amino acids	Other	9145097	8816228	8992847	8744029	13031345	14321500	13155961	13480823	28556512	27447295	27907266	29185454
31	L-Tyrosine	Amino acids	Other	5194166	5306296	5172633	5382620	4740798	4829050	4841764	4908980	19856129	20962892	20363623	21417762
32	N-Acetyl-L-Glutamic Acid	Amino acids	Other	160807.6	155438.6	1.14E-05	68482.1	113316.7	109915.4	125938.3	128574.3	338511.4	340730.7	336728.1	350663.8
33	N-Acetyl-L-Methionine	Amino acids	Other	276511.9	277199.9	276720.3	261727.1	95920.56	114224.9	80207.02	98395.99	104551.1	109320.6	111781.1	112086.5
34	N-Carbamoyl-Dl-Aspartic Acid	Amino	Other	322704.2	325861.4	327463	329878	683474.1	809150.5	693686	784488.3	8379203	8519189	8541953	8302856

		acids													
35	Nmda	Amino acids	Other	15351.99	5140.158	2751.596	4176.477	2742.612	2141.83	4573.721	4741.243	1860.7	3539.361	5211.45	6128.521
36	O-Acetylserine	Amino acids	Other	373041.1	397969.2	341552	348332.5	360415.5	346386	380329.1	343948.8	152345.2	151100.8	130807.3	122723.5
37	Se-Methylselenocysteine	Amino acids	Other	857117.6	465171.7	892035.2	519406.5	1080359	1309210	1708167	1337214	271993.3	245958.7	206183.3	196115.3
38	B-Cyano-L-Alanine	Amino acids	Other	457915.1	515646.2	470894.7	469380.4	508262.5	487249.5	502333	482680.1	963234.3	822494.7	914395.7	936122.4
39	1-Methyl-L-Histidine	Amino acids	Other	3909613	3514054	3216199	3309507	2801079	3181951	2869630	3108476	2406762	2540471	2158350	2279913
40	2-Amino-5-Ureidopentanoic Acid	Amino acids	Other	14546.9	4951.557	10333.42	15619.67	26628.32	47453.48	79622.98	74687.29	176922.8	351833.7	112633.6	290403.7
41	2-Chloro-Dl-Phenylalanine	Amino acids	Other	84290.63	75950.29	69568.64	91898.24	71433.54	78000.32	119616.7	98613.51	137311.4	140976	111053.8	209295.2
42	2-(Methylamino)Benzoic Acid	Amino acids	Other	11241067	11287218	11124519	11521343	11424236	12336514	12185969	12035204	17687401	17358523	17573018	18922407
4	3-Nitro-L-Tyrosine	Ami	Other	1.14E-	1.14E-	1.14E-	1.14E-	1.14E-	35688.	1.14E-	1.14E-	1.14E-	1.14E-	34911.	1.14E-

3		no acids		05	05	05	05	05	09	05	05	05	05	36	05
44	3-O-Methyldopa	Amino acids	Other	5061185	5315494	6452510	6460177	586244.1	3160710	513768	3046049	1.14E-05	1.14E-05	1.14E-05	1.14E-05
45	6-Amino-2-Oxohexanoate	Amino acids	Other	73577.63	75995.12	63556.69	79903.83	110819.7	120344.9	157614.7	98987.35	144287.1	135514.6	181383.1	133253.3
46	D(-)-2-Aminobutyric Acid	Amino acids	Other	1387833	1199358	1262347	1421343	755564.5	795378.9	709480.5	722714.4	123855.2	109831.2	132885.4	82419.01
47	D-Ornithine (Hydrochloride)	Amino acids	Other	13917.9	74174.6	64446.41	83482.43	79078.22	54734.63	56425.8	77878.03	1.14E-05	17816.94	1.14E-05	6508.951
48	D-Phenylalanine	Amino acids	Other	47087342	45559248	48470357	47884265	37111143	37355334	38072828	39154358	49271181	56633695	41055314	1.14E-05
49	D-Serine	Amino acids	Other	143918.2	159351.6	202415.5	71854.47	67683.85	341560.8	34050.49	292975.8	422293.6	71999.01	101882.7	101604.3
50	H-Gly-Pro-Oh	Amino acids	Other	329471.7	632745.4	355608.4	321467.6	76162.71	148770.2	163589.7	87144.71	137766.5	135790.2	140221.6	150703.9
51	H-Hoarg-Oh	Amino acids	Other	825090.5	750254.7	742044.7	778225.3	452747	461963.6	454585.6	489468.8	1.14E-05	209579.7	1.14E-05	1.14E-05

52	L-Arginine (Hydrochloride)	Amino acids	Other	8010649	7624911	8363903	2295001	6828876	6299561	6459688	6940721	14929678	14386726	14213858	13327196
53	L-Citrulline	Amino acids	Other	8788525	8736675	9098649	8978005	7091627	6803436	6492163	7087100	23661871	22646066	22192834	22020392
54	Leucyl-Phenylalanine	Amino acids	Other	55368.12	61755.41	54374.06	41928.99	88129.01	57764.17	113193.7	72160.18	163279.7	132370.5	153570.9	146990.1
55	L-Histidine	Amino acids	Other	963706.1	1144913	995297.4	814003.6	722788.8	722500.8	829217.2	753585	666367.3	739547.8	733413	726606.8
56	L-Lysine	Amino acids	Other	8666574	8505890	8619753	8800154	12379904	13273544	13017444	12804444	85209986	83176645	82566844	75406031
57	L-Methionine	Amino acids	Other	202738.6	252645.2	243146.9	238204.7	168268.1	184669.1	242786.8	147387.2	182799.9	169632.2	158582.7	189626.7
58	L-Nmma (Acetate)	Amino acids	Other	3124824	3113563	3215094	3172224	2053105	2427364	2548623	1999235	23661871	1.14E-05	1.14E-05	1.14E-05
59	L-Pipecolic Acid	Amino acids	Other	3909613	3514054	3216199	3309507	2801079	3181951	2869630	3108476	2406762	2540471	2158350	2279913
60	L-Proline	Amino	Other	2269621	2140849	1451740	1271693	1889890	2982154	1758965	2526597	3016894	3203024	2716488	1847856

		acids													
61	Nepsilon-Acetyl-L-Lysine	Amino acids	Other	500204.8	518328.6	510748.3	564751.1	399954.6	470852.1	457105.2	463201.6	112453	117448.4	107267	109097.3
62	Ng,Ng-Dimethylarginine Dihydrochloride	Amino acids	Other	305089.2	198355.5	238320.7	322873	301507.8	342264.3	312286.2	425220.9	529780.7	413771.4	285706.5	492919.1
63	Nsc 16590	Amino acids	Other	7758783	7780836	7636150	7437838	10167471	11031085	11440288	11530526	82421568	79738107	75487328	74721523
64	Phenylacetylglutamine	Amino acids	Other	172548.4	194954.1	222672	191328.1	57151.56	74368.4	79066.06	78861.11	94265.68	50725.88	59359.65	48090.57
65	Sdma	Amino acids	Other	200740.6	245744.5	194666.9	245440.2	162873.7	175442.4	166012.5	243676.5	159058	133128.6	131727.1	116543.4
66	Selenomethionine	Amino acids	Other	1886389	1816172	1709717	1259215	985355.5	968960.4	1118082	942861.8	279272	218849.9	272374.5	121186.2
67	Ureidopropionic Acid	Amino acids	Other	20004.97	33733.13	1.14E-05	38667.61	31035.81	25309.26	1.14E-05	1.14E-05	1.14E-05	1.14E-05	38615.56	1.14E-05
68	γ-Glu-Phe	Amino acids	Other	1585.361	1046.403	2749.248	1363.127	1.14E-05	1342.903	1.14E-05	1.14E-05	1443.796	5828.951	671.2914	2019.042
6	S-(5'-Adenosy)-L-	Ami	Other	37.066	41.407	33.860	33.737	36.596	37.916	29.429	30.168	3.1357	12.902	5.0633	3.2569

9	homocysteine	no Acids		44	99	49	44	45	51	46	64	38	33	8	15
70	Phe-His	Amino Acids	Other	1.14E-05	1.14E-05	1.14E-05	1.14E-05	1.14E-05	1.14E-05	1.14E-05	1.14E-05	1.14E-05	1.14E-05	1.14E-05	1.14E-05

Table S3. 43 differential metabolites of Amino acids in *L. chinense* fruits among three stages

Number	Name	Class I	Class II	Ratio of Metabolite Contents	
				CCF/GF	RRF/GF
1	L-Phenylalanyl-L-Leucine	Amino acids	Peptides	0.19	0.20
2	Pyruvic Acid	Amino acids	Peptides	19.57 ↑	1.53 ↑
3	γ-L-Glutamyl-L-Alanine	Amino acids	Peptides	2.01 ↑	4.96↑
4	Cyclo(Leu-Leu)	Amino acids	Dipeptide	0.71	0.22
5	Cyclo(Phe-Hpro)	Amino acids	Dipeptide	0.46	1.04
6	Cyclo(Tyr-Val)	Amino acids	Dipeptide	0.24	0.00
7	Dl-Tryptophan	Amino acids	Alpha Amino Acids	0.20	5.09
8	N'-Formylkynurenine	Amino acids	Alpha Amino Acids	0.42	0.14
9	(-)-Aspartic Acid	Amino acids	Other	1.06↑	2.95↑
10	2-Amino-5-Ureidopentanoic Acid	Amino acids	Other	5.02 ↑	4.08 ↑
11	3-O-Methyldopa	Amino acids	Other	0.31	0.00
12	4-Hydroxyisoleucine	Amino acids	Other	1.40↑	1.68 ↑
13	6-Amino-2-Oxohexanoate	Amino acids	Other	1.66 ↑	1.22 ↑

14	Ac-Ala-Oh	Amino acids	Other	1.18	0.04
15	D(-)-2-Aminobutyric Acid	Amino acids	Other	0.57	0.15
16	Dl-Asparagine	Amino acids	Other	1.14 ↑	2.07 ↑
17	D-Ornithine (Hydrochloride)	Amino acids	Other	1.14	0.09
18	H-Gly-Pro-Oh	Amino acids	Other	0.29	1.19
19	H-Hoarg-Oh	Amino acids	Other	0.60	0.11
20	L-Allothreonine	Amino acids	Other	1.54↑	2.16 ↑
21	L-Arginine (Hydrochloride)	Amino acids	Other	1.01↑	2.14 ↑
22	L-Aspartic Acid	Amino acids	Other	1.03↑	2.72↑
23	L-Citrulline	Amino acids	Other	0.77	3.29
24	Leucyl-Phenylalanine	Amino acids	Other	1.55↑	1.80 ↑
25	L-Glutamic Acid	Amino acids	Other	1.78 ↑	1.49↑
26	L-Glutamic Acid (Monosodium Salt)	Amino acids	Other	1.98 ↑	1.51↑
27	L-Lysine	Amino acids	Other	1.49 ↑	6.34 ↑
28	L-Methionine Sulfoxide	Amino acids	Other	1.60 ↑	1.32 ↑
29	L-Ornithine (Hydrochloride)	Amino acids	Other	1.17 ↑	2.98 ↑
30	L-Serine	Amino acids	Other	2.56↑	2.92 ↑
31	L-Threonine	Amino acids	Other	1.51↑	2.09 ↑
32	L-Tyrosine	Amino acids	Other	0.92	4.28
33	N-Acetyl-L-Glutamic Acid	Amino acids	Other	1.24↑	2.86 ↑
34	N-Acetyl-L-Methionine	Amino acids	Other	0.36	1.13
35	N-Carbamoyl-Dl-Aspartic Acid	Amino acids	Other	2.27↑	11.36↑
36	Nepsilon-Acetyl-L-Lysine	Amino acids	Other	0.86	0.25
37	Nsc 16590	Amino acids	Other	1.44↑	7.07 ↑
38	O-Acetylserine	Amino acids	Other	0.98	0.39
39	Phenylacetylglutamine	Amino acids	Other	0.37	0.87

40	S-(5'-Adenosy)-L-homocysteine	Amino acids	Other	0.92	0.18
41	Selenomethionine	Amino acids	Other	0.60	0.22
42	Se-Methylselenocysteine	Amino acids	Other	1.99	0.17
43	γ-Glu-Phe	Amino acids	Other	0.20	7.42

Table S4. 14 Amino Acid Metabolites Categories of *L. chinense* Fruit in KEGG database

First Category	Second Category	ko ID	Annotated numbers
Amino acid metabolism	Arginine biosynthesis	ko00220	5
	Alanine, aspartate and glutamate metabolism	ko00250	6
	Glycine, serine and threonine metabolism	ko00260	8
	Cysteine and methionine metabolism	ko00270	9
	Valine, leucine and isoleucine degradation	ko00280	1
	Valine, leucine and isoleucine biosynthesis	ko00290	3
	Lysine biosynthesis	ko00300	3
	Lysine degradation	ko00310	7
	Arginine and proline metabolism	ko00330	4
	Histidine metabolism	ko00340	5
	Tyrosine metabolism	ko00350	4
	Phenylalanine metabolism	ko00360	7
	Tryptophan metabolism	ko00380	4
	Phenylalanine, tyrosine and tryptophan biosynthesis	ko00400	5

Total	71

Table S5. 170 genes associated with the synthesis of amino acids in *L. chinense* fruit

Gene	GF	CCF	RRF
TRINITY_DN35763_c0_g3	14.30069	19.66731	57.714
TRINITY_DN35763_c0_g2	0.360914	0.224027	0
TRINITY_DN61339_c0_g1	0.236234	0.230861	0
TRINITY_DN37253_c3_g1	13.03806	18.02621	127.6631
TRINITY_DN32384_c5_g12	0.356315	0	0.193648
TRINITY_DN1310_c0_g1	0.159243	0.155838	0
TRINITY_DN38139_c5_g1	42.85564	73.47751	180.829
TRINITY_DN16658_c0_g1	0.021554	0.779997	0
TRINITY_DN36643_c0_g1	24.64791	25.41952	87.90111
TRINITY_DN36595_c1_g3	0.068824	0.40644	0.076687
TRINITY_DN78999_c0_g1	0.251163	0	0
TRINITY_DN1203_c0_g1	0.058385	0.176304	0.02332
TRINITY_DN26864_c0_g2	0	0.352967	0
TRINITY_DN42541_c0_g1	0	0.725503	0
TRINITY_DN91154_c0_g1	0.143188	0.258063	0.069747

TRINITY_DN14366_c0_g1	0	0.733937	0
TRINITY_DN56898_c0_g1	0.595668	0.499942	0
TRINITY_DN17685_c0_g1	0.422883	0.203856	0
TRINITY_DN13239_c0_g2	0	0	0
TRINITY_DN33848_c0_g1	136.4435	306.4111	193.2095
TRINITY_DN39380_c2_g3	2.827427	3.558842	5.872666
TRINITY_DN36595_c1_g2	82.54121	93.39121	340.5686
TRINITY_DN24621_c0_g1	0.41384	0	0
TRINITY_DN41382_c0_g1	7.930551	10.26326	12.3811
TRINITY_DN46599_c0_g1	0.509748	0.097109	0
TRINITY_DN47803_c0_g1	0.259081	0.08741	0
TRINITY_DN11990_c0_g1	0.206688	0.069886	0
TRINITY_DN10640_c0_g1	0.291985	0	0
TRINITY_DN35286_c0_g2	3.703988	2.023422	4.912294
TRINITY_DN35286_c0_g1	2.700981	17.40956	65.39167
TRINITY_DN16259_c0_g1	0.08079	0.235337	0
TRINITY_DN97953_c0_g1	0.247465	0.067324	0
TRINITY_DN69246_c0_g1	0	0.210004	0
TRINITY_DN11330_c0_g1	0.256361	0.046194	0.101596
TRINITY_DN11769_c0_g1	0.32564	0	0
TRINITY_DN41833_c0_g8	5.089227	2.027907	5.267743
TRINITY_DN41833_c0_g1	46.37867	14.10568	29.94414
TRINITY_DN41833_c0_g7	1.053478	2.652567	1.931173
TRINITY_DN41833_c0_g4	2.745101	1.523894	2.956361
TRINITY_DN104198_c0_g1	0	0.628703	0
TRINITY_DN38259_c0_g6	0.786041	2.255359	2.590724

TRINITY_DN38259_c0_g7	53.4495	55.76256	136.7459
TRINITY_DN38259_c0_g3	49.60606	4.342195	1.309863
TRINITY_DN33430_c1_g1	29.26361	26.18532	35.40368
TRINITY_DN33430_c1_g5	2.655109	7.288107	3.357971
TRINITY_DN20410_c0_g1	0.006834	0.513683	0
TRINITY_DN85028_c0_g1	0.095446	0.111403	0
TRINITY_DN7801_c0_g1	0.039673	0.235531	0.04305
TRINITY_DN4614_c0_g1	0.140004	0.136384	0
TRINITY_DN38906_c0_g5	0.286316	0.100537	1.653423
TRINITY_DN38906_c0_g2	9.541433	17.63925	21.19899
TRINITY_DN38906_c0_g1	4.528028	19.55347	9.857664
TRINITY_DN40927_c1_g7	0.457075	0.106525	0.123248
TRINITY_DN40927_c1_g1	71.47659	49.70748	107.8007
TRINITY_DN32942_c3_g2	0.159813	0.202778	1.004591
TRINITY_DN41425_c2_g1	4.575346	2.685033	5.174636
TRINITY_DN29237_c0_g2	0.471005	0	0
TRINITY_DN41425_c1_g1	31.22457	9.172732	19.40405
TRINITY_DN41425_c1_g2	16.13127	5.154875	12.44066
TRINITY_DN41425_c1_g3	0.097713	0.306288	0.298411
TRINITY_DN19150_c0_g1	0.496004	0	0
TRINITY_DN8614_c0_g1	0.341516	0.032148	0.074816
TRINITY_DN81032_c0_g1	0	0.278885	0
TRINITY_DN66351_c0_g1	0	0	0.397517
TRINITY_DN53330_c0_g1	0.109996	0.06024	0
TRINITY_DN53142_c0_g1	0.097254	0.058172	0
TRINITY_DN42467_c0_g1	0	0.182524	0.199753

TRINITY_DN37090_c0_g1	0.835318	0.490512	0.157767
TRINITY_DN17461_c0_g1	0.066639	0.129518	0.071995
TRINITY_DN37090_c1_g1	44.93999	37.77225	60.15331
TRINITY_DN94027_c0_g1	0.403461	0.187577	0
TRINITY_DN54207_c0_g1	0.530127	0	0
TRINITY_DN104509_c0_g1	0.144067	0.141022	0
TRINITY_DN3548_c0_g1	0.211522	0.04566	0.051292
TRINITY_DN34998_c3_g5	0.368829	0.793036	1.424707
TRINITY_DN8521_c0_g1	0.700733	0	0
TRINITY_DN90947_c0_g1	0.273531	0	0.09245
TRINITY_DN1310_c0_g1	0.159243	0.155838	0
TRINITY_DN631_c0_g1	0.309891	0.074055	0.083102
TRINITY_DN42215_c1_g10	0.489121	1.671163	6.92354
TRINITY_DN32630_c0_g1	4.386614	0.173849	0.099873
TRINITY_DN35354_c4_g9	0.444148	0	0.524592
TRINITY_DN36267_c0_g1	8.748174	6.067656	4.305098
TRINITY_DN34887_c6_g2	38.23575	66.54605	253.1156
TRINITY_DN24050_c0_g1	0.119977	0.070089	0.037015
TRINITY_DN37483_c3_g1	0.808805	0.346229	0.327069
TRINITY_DN45236_c0_g1	0.211832	0.250605	0
TRINITY_DN36504_c1_g2	1.99188	0	0
TRINITY_DN60349_c0_g1	0.651534	0.153924	0
TRINITY_DN97093_c0_g1	0	0.446185	0
TRINITY_DN32636_c1_g1	36.47047	36.14585	49.19805
TRINITY_DN66675_c0_g1	0.185332	0.109903	0
TRINITY_DN91791_c0_g1	0.039136	0.194967	0

TRINITY_DN40711_c3_g3	28.22491	30.84369	29.365
TRINITY_DN98580_c0_g1	0.122075	0	0
TRINITY_DN35416_c2_g1	5.001251	4.829451	7.326925
TRINITY_DN35416_c2_g3	6.573523	20.14844	32.07001
TRINITY_DN35416_c2_g6	0.031598	0.179492	0.261616
TRINITY_DN35416_c2_g7	0	0.094671	0.354851
TRINITY_DN89654_c0_g1	0.438983	0.151914	0
TRINITY_DN54089_c0_g1	0	0.484026	0
TRINITY_DN16031_c0_g1	0.119346	0.044659	0
TRINITY_DN38775_c1_g1	15.25055	22.85191	13.78925
TRINITY_DN10760_c0_g1	0.255854	0.408669	0
TRINITY_DN16630_c0_g1	0.312939	0.120049	0
TRINITY_DN52927_c0_g1	0.065184	0.079321	0
TRINITY_DN76400_c0_g1	0	0.169147	0
TRINITY_DN88055_c0_g1	0.389363	0	0
TRINITY_DN32749_c0_g1	10.50416	33.60552	27.26454
TRINITY_DN41801_c5_g1	117.4132	91.7656	93.05411
TRINITY_DN41801_c5_g4	0.106753	0.137052	0.448
TRINITY_DN41801_c5_g2	0	0.043975	0.198811
TRINITY_DN41801_c5_g3	0.572425	0.44742	1.665263
TRINITY_DN105551_c0_g1	0	0.685213	0
TRINITY_DN106967_c0_g1	0.554963	0	0
TRINITY_DN88235_c0_g1	0.253119	0	0
TRINITY_DN66722_c0_g1	0.545302	0	0.141125
TRINITY_DN36252_c0_g3	1.376128	1.126855	2.241162
TRINITY_DN36621_c2_g2	7.399235	212.2863	19.39415

TRINITY_DN36621_c2_g5	31.09363	6.00943	28.29583
TRINITY_DN36621_c2_g4	21.37777	5.976238	33.04103
TRINITY_DN36621_c2_g6	0.066645	1.30222	0.11842
TRINITY_DN38526_c0_g1	30.49956	25.23787	37.26628
TRINITY_DN38526_c0_g2	4.147011	6.857783	5.533174
TRINITY_DN34029_c4_g4	13.88468	19.81928	18.69366
TRINITY_DN41763_c3_g1	0.260683	0.029409	0.011406
TRINITY_DN41012_c0_g2	42.55749	24.51458	35.56713
TRINITY_DN41012_c0_g1	35.95527	40.60013	27.27121
TRINITY_DN9875_c0_g1	0.477557	0	0
TRINITY_DN107450_c0_g1	0.126579	0.119517	0
TRINITY_DN41012_c1_g2	15.33382	18.81056	26.58156
TRINITY_DN41012_c1_g1	56.78964	76.28096	65.26014
TRINITY_DN78819_c0_g1	0.219107	0.318525	0
TRINITY_DN64407_c0_g1	0	0.411197	0
TRINITY_DN61862_c0_g1	0.202837	0.236858	0
TRINITY_DN46045_c0_g1	0.121356	0.297649	0
TRINITY_DN46316_c0_g1	0.147004	0.140792	0
TRINITY_DN81329_c0_g1	0	0	0.46356
TRINITY_DN76107_c0_g1	0.11377	0.110699	0
TRINITY_DN47195_c0_g1	0	0.129519	0
TRINITY_DN83465_c0_g1	0.349411	0	0
TRINITY_DN41835_c1_g1	87.47934	133.1898	233.5409
TRINITY_DN69673_c0_g1	0	0.212286	0.240574
TRINITY_DN74791_c0_g1	0.134085	0.071244	0
TRINITY_DN3201_c0_g1	0.181234	0.290901	0

TRINITY_DN75858_c0_g1	0	0.694813	0
TRINITY_DN4183_c0_g1	0	0	0.217529
TRINITY_DN45720_c0_g1	0.45408	0.142698	0
TRINITY_DN47377_c0_g1	0.78325	0	0
TRINITY_DN77528_c0_g1	0.280401	0.301271	0
TRINITY_DN22481_c0_g2	0.290846	0.057664	0
TRINITY_DN22481_c0_g1	0.232639	0	0
TRINITY_DN98815_c0_g1	0.217328	0.206968	0
TRINITY_DN52473_c0_g1	0.047491	0.093285	0
TRINITY_DN95146_c0_g1	0.20246	0	0.072321
TRINITY_DN53269_c0_g1	0.156308	0.186718	0
TRINITY_DN90354_c0_g1	0.326744	0.247525	0
TRINITY_DN80009_c0_g1	0.058618	0.232308	0
TRINITY_DN56525_c0_g1	0	0.322853	0
TRINITY_DN52880_c0_g1	0.09132	0	0
TRINITY_DN76392_c0_g1	0.13558	0.130652	0
TRINITY_DN75138_c0_g1	0.127507	0	0.070866
TRINITY_DN33197_c0_g1	12.58525	21.06212	45.42791
TRINITY_DN8958_c0_g1	0	0.670847	0
TRINITY_DN98159_c0_g1	0.027678	0.20223	0
TRINITY_DN97814_c0_g1	0	0.122757	0
TRINITY_DN100188_c0_g1	0.216467	0.212039	0
TRINITY_DN60197_c0_g1	1.294156	0	0
TRINITY_DN46527_c0_g1	0.399455	0	0
TRINITY_DN80936_c0_g1	0.142565	0.04828	0

Table S6. 20 Unigenes in differential Amino acid metabolism of *L. chinense* fruits

Number	Name	Transcript No.	Pathway
1	*AASS1*	TRINITY_DN41801_c5_g1	Lysine biosynthesis (ko00300)
2	*AO1*	TRINITY_DN32636_c1_g1	Arginine biosynthesis (ko00220)
3	*AST1*	TRINITY_DN37253_c3_g1	Arginine biosynthesis (ko00220)
4	*AST2*	TRINITY_DN38139_c5_g1	
5	*AST3*	TRINITY_DN33848_c0_g1	
6	*AST4*	TRINITY_DN36595_c1_g2	
7	*dat1*	TRINITY_DN41012_c1_g1	D-Amino acid metabolism (ko00470)
8	*GLT1*	TRINITY_DN41835_c1_g1	Alanine, aspartate and glutamate metabolism (ko00250)
9	*ltaE1*	TRINITY_DN35286_c0_g1	Glycine, serine and threonine metabolism (ko00260)
10	*OTC1*	TRINITY_DN35416_c2_g3	Arginine biosynthesis (ko00220)
11	*PK1*	TRINITY_DN40291_c2_g4	Alanine, aspartate and glutamate metabolism (ko00250)
12	*PK2*	TRINITY_DN39863_c4_g1	
13	*PK3*	TRINITY_DN40975_c3_g6	
14	*racD1*	TRINITY_DN36539_c2_g3	Alanine, aspartate and glutamate metabolism (ko00250)
15	*SHMT1*	TRINITY_DN41833_c0_g1	Glycine, serine and threonine metabolism (ko00260)
16	*SHMT2*	TRINITY_DN38259_c0_g7	
17	*SHMT3*	TRINITY_DN40927_c1_g1	
18	*SHMT4*	TRINITY_DN41425_c1_g2	
19	*TAT1*	TRINITY_DN34887_c6_g2	Tyrosine metabolism (ko00350)
20	*thrC1*	TRINITY_DN37090_c1_g1	Glycine, serine and threonine metabolism (ko00260)

Table S7. FPKM of 20 Unigenes

Number	Name	GF1	GF2	GF3	CCF1	CCF2	CCF3	RRF1	RRF2	RRF3
1	*AASS1*	152.6138	147.0753	113.8002	115.0789	83.4797	76.7382	110.0547	62.93642	106.1712
2	*AO1*	75.86206	89.01595	56.41177	47.30619	28.05979	33.07157	51.06203	51.47684	45.05528
3	*AST1*	16.96942	18.8756	9.595148	32.66753	7.048748	14.36236	141.9272	101.758	139.3042
4	*AST2*	129.655	145.955	101.7829	114.1505	48.08719	58.19489	194.0077	145.1408	203.3384
5	*AST3*	162.4929	162.9715	198.8277	282.0417	340.6293	296.5623	170.7904	214.3004	194.5378
6	*AST4*	220.5559	193.0171	110.8828	138.7311	56.51064	84.93188	321.4973	230.9448	469.2637
7	*dat1*	322.6722	257.4662	214.5103	98.00777	60.70644	70.12867	79.10584	39.97098	76.70359
8	*GLT1*	202.5086	162.2302	160.8024	133.1449	131.9448	134.4797	157.8187	298.27	244.534
9	*ltaE1*	16.10384	6.451403	9.888885	21.25919	13.1538	17.8157	81.54462	54.7784	59.852
10	*OTC1*	17.96447	13.78805	16.32228	33.82374	11.0664	15.5552	36.68039	21.23129	38.29834
11	*PK1*	59.23181	53.82451	47.11644	57.46207	72.18065	76.64111	50.836	23.57321	55.28782
12	*PK2*	88.92713	102.1751	78.43274	115.0117	39.48084	50.50912	186.277	210.8219	167.4662
13	*PK3*	60.25694	53.52007	48.57114	46.55896	17.0262	28.3657	110.936	103.129	117.831
14	*racD1*	14.30571	20.0977	9.107924	11.60845	1.588608	2.980527	8.501737	5.289137	12.42813
15	*SHMT1*	111.0096	85.54068	53.45462	18.24355	10.93716	13.13634	30.76069	31.83781	27.23391
16	*SHMT2*	58.27538	45.9942	50.137	90.3009	36.53949	40.4473	126.968	126.5698	156.6997
17	*SHMT3*	56.63863	49.56586	45.34662	88.13912	27.48684	33.49647	109.0597	90.37258	123.9698
18	*SHMT4*	202.2611	149.5932	108.087	7.320887	3.487914	4.655825	12.43183	6.158461	18.73168
19	*TAT1*	74.49815	91.96102	42.74343	105.2181	31.07354	63.34652	227.2347	314.8113	217.3007
20	*thrC1*	59.30665	58.59351	45.41312	48.10458	26.5941	38.61806	61.43536	60.74019	58.28438

Table S8. Primers and Sequences For RT-qPCR

Number	Gene name	Primer Sequence (5′–3′) (Forward/Reverse)	GenBank No.	Product length(bp)	Transcript No. (Unigene)
1	*AASS1*	F: AGCCCATCACGGAAGTCT<br>R: TTTCCTCCCGTTAGCATT	PV391917	112	TRINITY_DN41801_c5_g1
2	*AO1*	F: AAGAGGAAGTTGGGCATGTAA<br>R: TAAGCCGTAGCCAGAAGTTTG	PV391915	107	TRINITY_DN32636_c1_g1
3	*AST1*	F: TCTGGTGGGTCCTGGTGA<br>R: ACATCCCGCTTCCTTACA	PV391906	184	TRINITY_DN37253_c3_g1
4	*AST2*	F: CGACCCATGTACTCCAACC<br>R: GCTTCTGTCTCACGCTCTTT	PV391907	136	TRINITY_DN38139_c5_g1
5	*AST3*	F: CCTTGAACCGTCGTCCTC<br>R: GGGCTGGTATCTTTATTGTATG	PV391908	150	TRINITY_DN33848_c0_g1
6	*AST4*	F: GAGCGTGTTGGTGCCCTTAG<br>R: TTCGTCGTACATTTGTCTGTCCTT	PV391909	162	TRINITY_DN36595_c1_g2
7	*dat1*	F: CTGGGTGCTGGTTTATGAT<br>R: ACCTGACTGTGGCTGTATG	PV391918	198	TRINITY_DN41012_c1_g1
8	*OTC1*	F: GCTCGTGTTCTGTCTGGCTAT<br>R: TTGGCAAGGATGGTTGTAGTC	PV391916	132	TRINITY_DN35416_c2_g3
9	*PK1*	F: ATCACGGACTTGTTGCTT<br>R: TCTTGCCCTTCCTTTAGC	PV391919	112	TRINITY_DN40291_c2_g4
10	*PK2*	F: TCACAGGAAGCGAAACAA<br>R: CCCAGCTAAAGTGGCAGA	PV391920	98	TRINITY_DN39863_c4_g1
11	*PK3*	F: AGGCACAGTTTGGTGTATCGG<br>R: GTTGGCATAGCCCTTTCGTG	PV391921	133	TRINITY_DN40975_c3_g6
12	*racD1*	F: ATAAGGCGACTATGGAACAC	PV391922	169	TRINITY_DN36539_c2_g3

Table S8. Primers and Sequences For RT-qPCR

Number	Gene name	Primer Sequence (5′–3′) (Forward/Reverse)	GenBank No.	Product length(bp)	Transcript No. (Unigene)
		R: TCTGGAGGTAACAGATCACG			
13	*SHMT2*	F: GGTGGCAACGAATACATC<br>R: TCCAATCCCATAATCCTATC	PV391910	176	TRINITY_DN38259_c0_g7
14	*SHMT3*	F: GGCTGTAACGATCACCTTG<br>R: ATCTCAGACATCTTGAACCC	PV391911	159	TRINITY_DN40927_c1_g1
15	*SHMT4*	F: TTGATTGTCGCTGGTGCT<br>R: GGACTTGTGGGTTGTGGTAG	PV391912	186	TRINITY_DN41425_c1_g2
16	*TAT1*	F: TTATCCCGTGAACTTCCAGAC<br>R: TAGAACCAGGGCGAGCCAAAA	PV391914	109	TRINITY_DN34887_c6_g2
17	*thrC1*	F: GTCACTGCTGAGTTGCCTAT<br>R: TCCCACCAGGAACTATCAC	PV391913	136	TRINITY_DN37090_c1_g1
18	*GAPDH*	F: GCTGTTGCGAGCATGGTG<br>R: GACAGCCCCTCAAACTCGTC	XM_060314892.1	112	TRINITY_DN39099_c1_g11
